# Single-cell transcriptomics reveals cell-type-specific diversification in human heart failure

**DOI:** 10.1038/s44161-022-00028-6

**Published:** 2022-03-16

**Authors:** Andrew L. Koenig, Irina Shchukina, Junedh Amrute, Prabhakar S. Andhey, Konstantin Zaitsev, Lulu Lai, Geetika Bajpai, Andrea Bredemeyer, Gabriella Smith, Cameran Jones, Emily Terrebonne, Stacey L. Rentschler, Maxim N. Artyomov, Kory J. Lavine

**Affiliations:** 1 Center for Cardiovascular Research, Department of Medicine, Cardiovascular Division, Washington University School of Medicine, St. Louis, MO, USA.; 2 Department of Pathology and Immunology, Washington University School of Medicine, St. Louis, MO, USA.; 3 Department of Developmental Biology, Washington University School of Medicine, St. Louis, MO, USA.

## Abstract

Heart failure represents a major cause of morbidity and mortality worldwide. Single-cell transcriptomics have revolutionized our understanding of cell composition and associated gene expression. Through integrated analysis of single-cell and single-nucleus RNA-sequencing data generated from 27 healthy donors and 18 individuals with dilated cardiomyopathy, here we define the cell composition of the healthy and failing human heart. We identify cell-specific transcriptional signatures associated with age and heart failure and reveal the emergence of disease-associated cell states. Notably, cardiomyocytes converge toward common disease-associated cell states, whereas fibroblasts and myeloid cells undergo dramatic diversification. Endothelial cells and pericytes display global transcriptional shifts without changes in cell complexity. Collectively, our findings provide a comprehensive analysis of the cellular and transcriptomic landscape of human heart failure, identify cell type-specific transcriptional programs and disease-associated cell states and establish a valuable resource for the investigation of human heart failure.

Single-cell and single-nucleus RNA sequencing (scRNA-seq and snRNA-seq, respectively) represent powerful new tools to identify cell types and their respective transcriptional signatures that reside within healthy and diseased tissues. Before the development of these technologies, our understanding of the cells that comprise human tissues and organs was restricted to routine histology and immunostaining analyses performed many decades ago. The rapid deployment of single-cell sequencing has revolutionized the field and resulted in the identification of previously unrecognized cell populations, including disease-specific cell states across a wide range of structures, including the brain, lung, liver, kidney and various malignancies^1-5^.

Heart failure represents a major cause of morbidity and mortality worldwide and imparts large costs on healthcare systems ($30–50 billion year^−1^ in the United States)^6,7^. Despite advancements in patient care, heart failure remains prevalent (lifetime risk of 20–45%) and portends 5-year morality rates approaching 50%, highlighting the clinical need to develop new therapies^8^. While bulk RNA-seq has yielded important insights into disease mechanisms that contribute to heart failure pathogenesis^9^, cell-specific information is lost and much remains to be learned regarding the roles of individual cell types. Identification of cell-specific disease-associated programs may provide the insights and opportunities necessary to develop new approaches for heart failure.

Recently, scRNA-seq and snRNA-seq was performed on healthy human heart tissue^10,11^. These studies yielded new information pertaining to common and rare cell populations within the healthy heart. Cardiomyocytes, fibroblasts, endothelial cells, pericytes, smooth muscles cells, myeloid cells, lymphoid cells, adipocytes and neural cells were readily identified and analyzed across anatomical sites. Distinct transcriptional states of atrial and ventricular cardiomyocytes were identified and validated using RNA in situ hybridization. Notable diversity was also observed among perivascular and immune cell types, including transcriptional signatures specific to different regions of heart.

At present, little is understood regarding the functional relevance of cell diversity within major human cardiac cell populations. Furthermore, the impact of human cardiac disease on cell composition remains to be rigorously investigated. While extensive work has been carried out in mouse models of heart failure, current scRNA-seq datasets exploring human heart failure are small and lack the sample size necessary to elucidate the impact of disease on common and rare cardiac cell types^12-21^.

Herein, we performed snRNA-seq and scRNA-seq on a large cohort of heart specimens obtained from healthy individuals and patients with chronic heart failure. We identified 15 major cardiac cell types from 45 individuals and explored the extent of cell diversity within each of these populations. Unsupervised clustering, differential gene expression and trajectory analyses revealed cell type-specific transcriptional programs and emergence of disease-associated cell states in the context of heart failure. We uncovered cell-specific influences of age on gene expression that differed based on disease state. Our data provide a comprehensive analysis of the cellular and transcriptomic landscape of the healthy and failing human heart and will serve as a valuable resource to the scientific community.

## Results

### snRNA-seq and scRNA-seq reveal the cellular landscape of the human heart.

**Extended Data Fig. 1 ∣:**
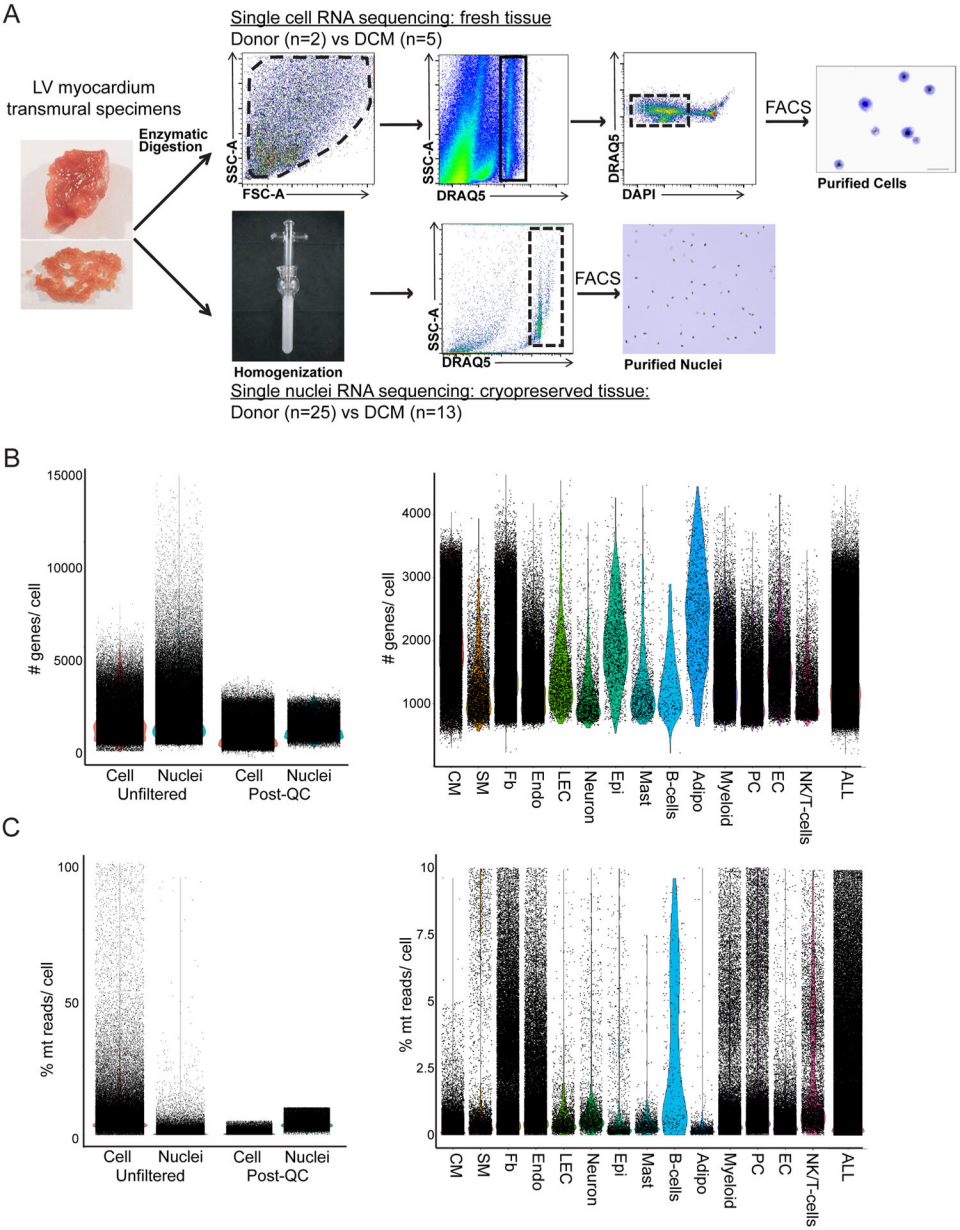
Sample processing and QC Plots. A, Diagram of tissue processing and flow cytometry cell sorting strategies for single cell RNA sequencing (top) and single-nucleus RNA sequencing (bottom). Plots are representative density plots (blue indicates low density while yellow indicates higher density. B, Violin plots of the number of genes per cell/nuclei split by sequencing technology for the integrated Seurat object before and after QC filtering (left) and after QC filtering split by cell type (right). C, Violin plots of the percent mitochondrial reads per cell/nuclei split by sequencing technology for the integrated Seurat object before and after QC filtering (left) and after QC filtering split by cell type (right).

**Fig. 1 ∣:**
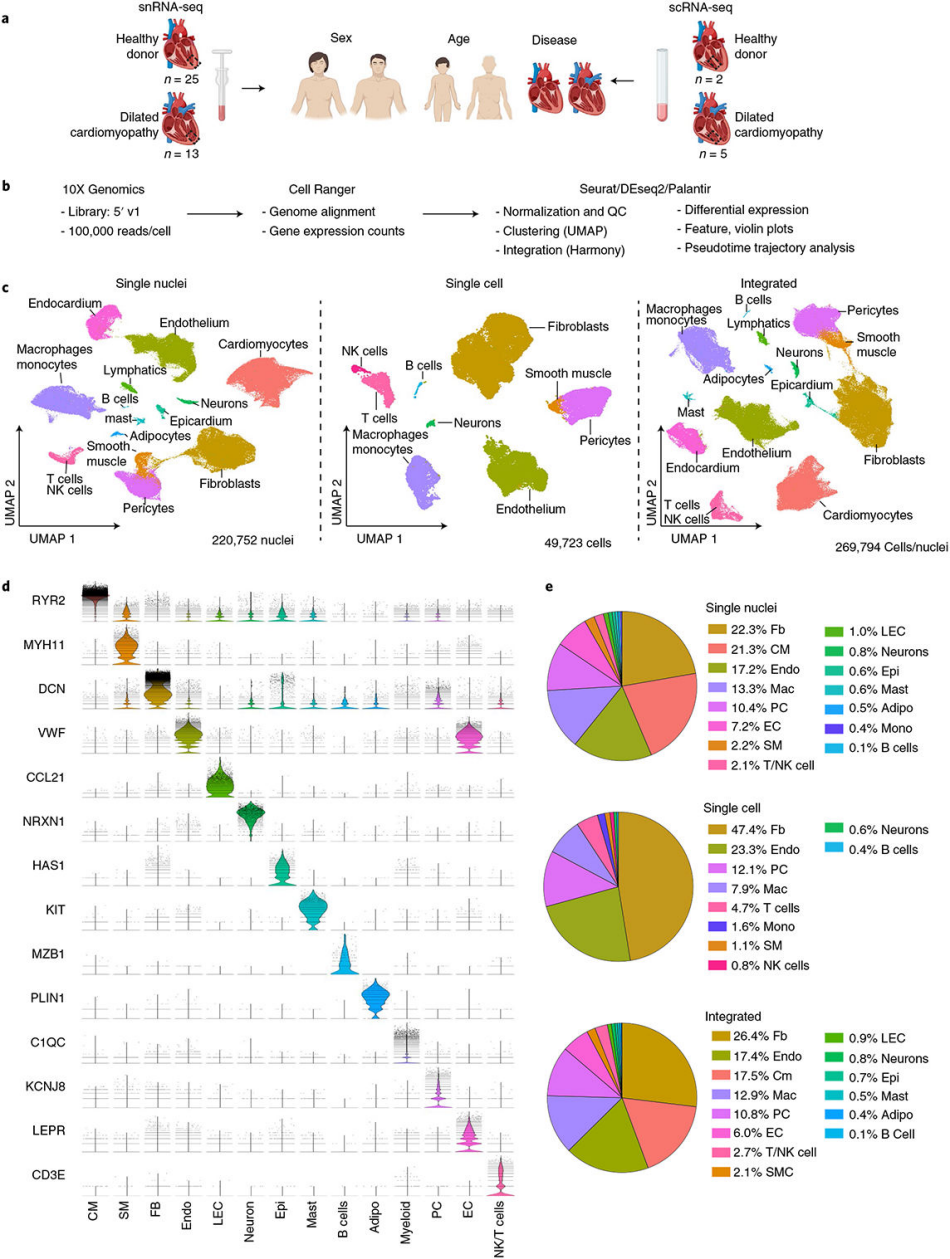
Cellular composition of the healthy and failing human heart. **a**, Schematic depicting design of the snRNA-seq and scRNA-seq experiments. Transmural sections were obtained from the apical anterior wall of the left ventricle during donor heart procurement, LVAD implantation or heart transplantation for comparison of disease, sex and age (snRNA-seq, *n* = 25 donor control, *n* = 13 dilated cardiomyopathy; scRNA-seq, *n* = 2 donor control, *n* = 5 dilated cardiomyopathy). Dashed box indicates location where sample was collected. LVAD, left ventricular assist device. **b**, The analysis pipeline included tissue processing and single-cell barcoded library generation (10X Genomics 5′ v1 kit), sequence alignment (Cell Ranger) and further analysis using R and Python packages (Seurat, Harmony, DEseq2, Palantir, ClusterProfiler and Enrichr). **c**, Unsupervised Uniform Manifold Approximation and Projection (UMAP) clustering of 220,752 nuclei, 49,723 cells and an integrated dataset combining snRNA-seq and scRNA-seq data after QC and data filtering using Harmony integration. **d**, Violin plots generated from the integrated dataset displaying characteristic marker genes of each identified cell population. **e**, Pie chart showing the proportion of cells within the snRNA-seq, scRNA-seq and integrated datasets.

To define the cellular and transcriptional landscape of the healthy and failing human heart, we obtained left ventricular (LV) cardiac tissue specimens from 28 non-diseased donors (donation after brain death) and 17 individuals with dilated (non-ischemic) cardiomyopathy (DCM). Non-diseased tissues were acquired from prospective donor hearts with normal LV function that were not used for transplantation due to the lack of a suitable recipient. DCM tissue was obtained from individuals undergoing implantation of an LV assist device or explanted hearts collected at the time of transplantation. Transmural myocardial samples from the apical and anterior segments of the LV were processed for either snRNA-seq (*n* = 38) or scRNA-seq (*n* = 7) using the 10X Genomics 5′ Single Cell platform (Fig. 1a, Extended Data Fig. 1 and Supplementary Table 1).

**Extended Data Fig. 2 ∣:**
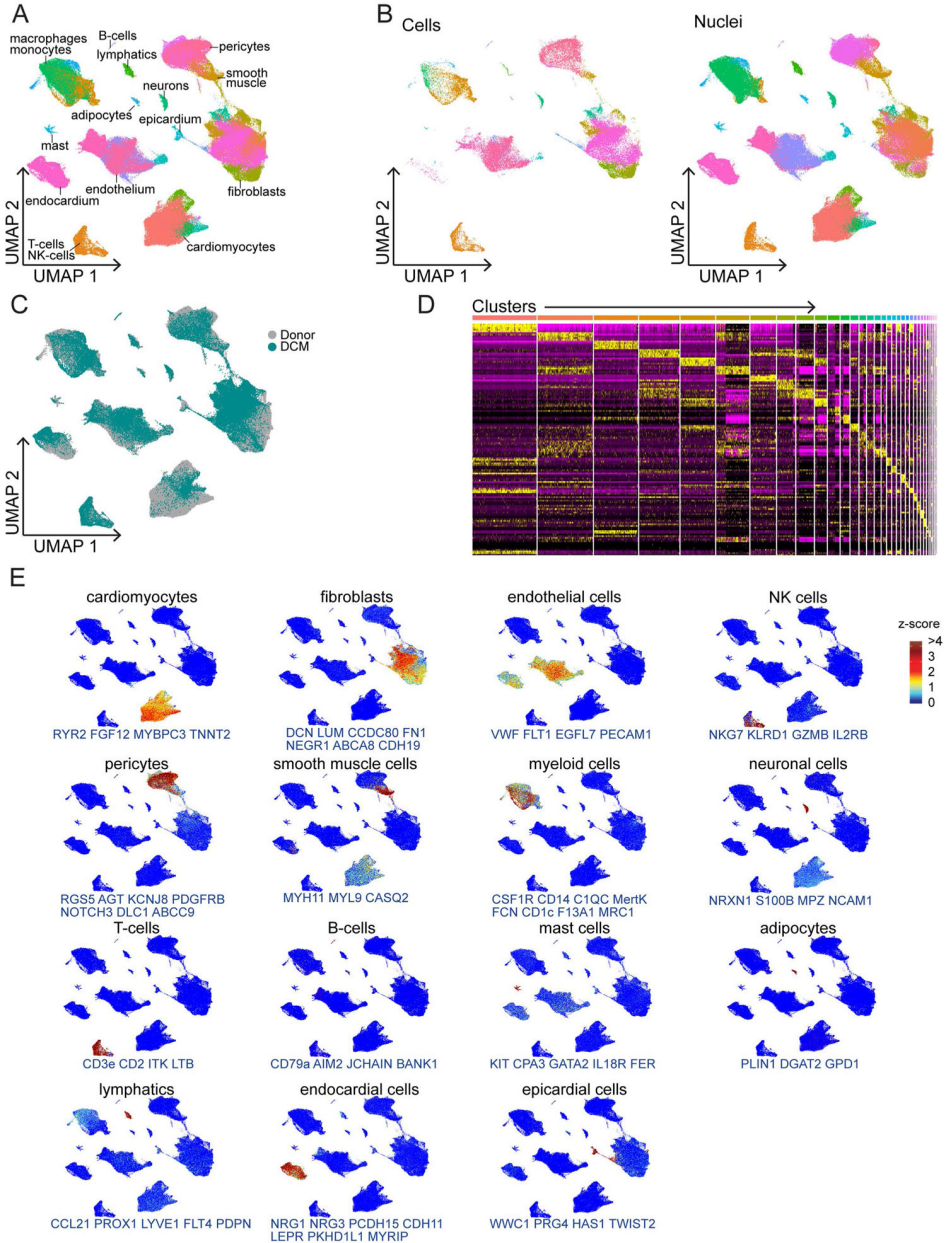
Integration of single cell RNA sequencing and single-nucleus RNA sequencing data allows for combined analysis of samples from different technologies. A, UMAP projection showing unsupervised clustering of the integrated dataset. B, UMAP projection split by technology. C, UMAP projection colored by disease state. D, Heat map of the top 10 genes by log2FC enriched in each cluster. E, Z-score feature plots for transcriptional signatures enriched in each cell type. Genes used for cell type identification (blue) were selected based on enrichment from Seurat differential expression analysis.

**Extended Data Fig. 3 ∣:**
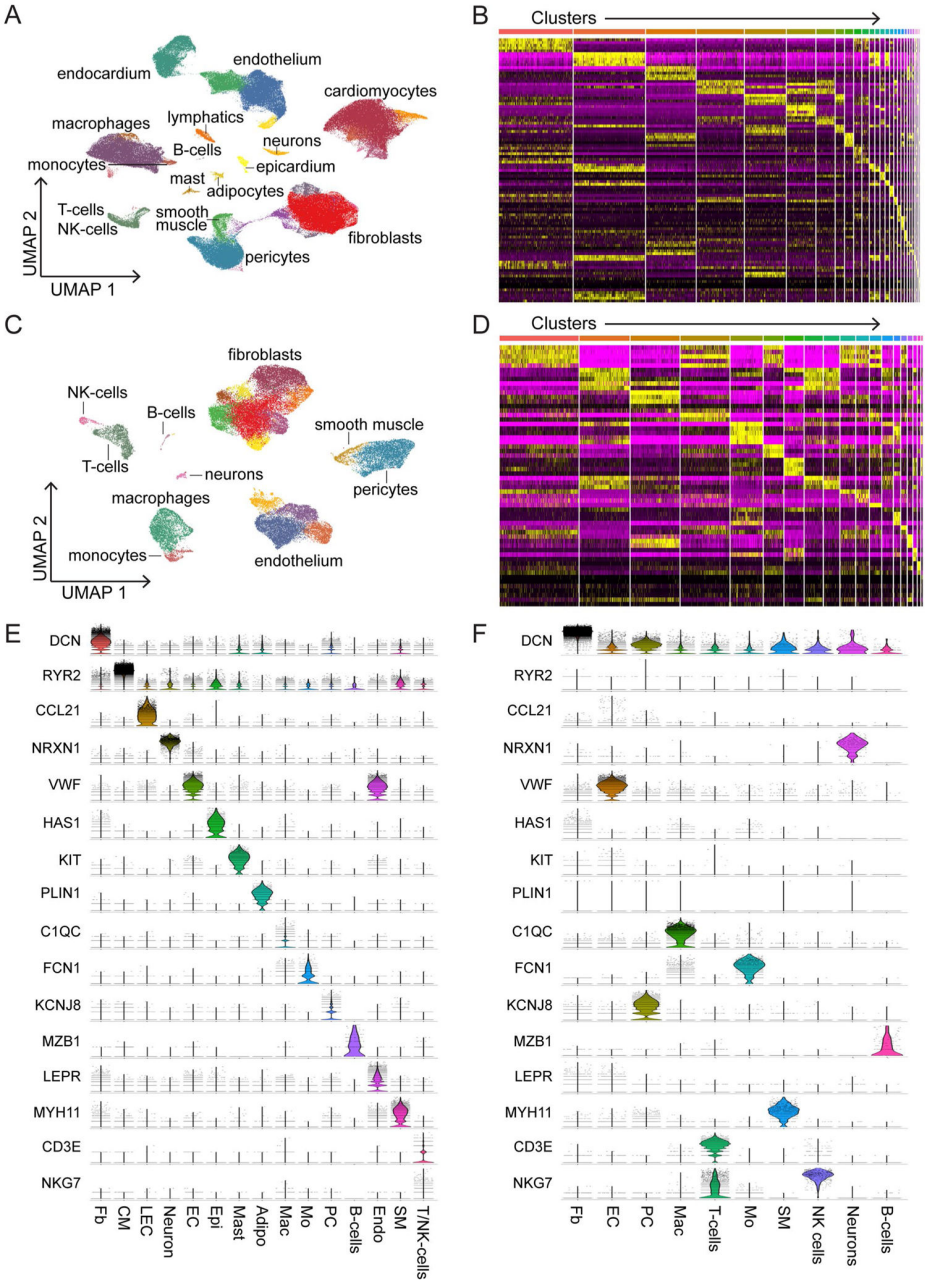
Single-cell and nucleus RNA sequencing identifies major cell populations within the LV myocardium. A, UMAP projection showing unsupervised clustering of single-nucleus RNA sequencing data. B, Heatmap of the top 10 genes by log2FC enriched in each cluster within single-nucleus RNA sequencing dataset. C, UMAP projection showing unsupervised clustering of single cell RNA sequencing data. D, Heatmap of the top 10 genes by log2FC enriched in each cluster within single cell RNA sequencing dataset. E-F, Violin plots split by cluster displaying the expression of characteristic cell marker genes in the single-nucleus RNA sequencing (E) and single cell RNA sequencing (F) datasets.

Single-nucleus and single-cell libraries were sequenced, aligned to the human reference genome, filtered for quality control (QC). Unsupervised clustering, integration and differential expression analysis performed using Harmony and Seurat (Fig. 1b, Extended Data Fig. 1 and Supplementary Tables 2 and 3). Following QC, nuclei samples had average gene and feature counts per cell of 2,849 and 1,496, respectively, whereas those counts for cells were 4,893 and 1,966, respectively. The final integrated dataset consisted of 220,752 nuclei and 49,723 cells representative of 15 major cell types (Fig. 1c). Cell identities were validated by expression of cell-specific marker genes (Fig. 1d) and transcriptional signatures (Extended Data Figs. 2 and 3). Cell types identified in both snRNA-seq and scRNA-seq datasets included fibroblasts, endothelial cells, myeloid cells, pericytes, smooth muscle cells, T cells and natural killer (NK) cells, neurons/glia and B cells. A notable benefit of snRNA-seq is the ability to obtain reads from additional cell types that are not efficiently recovered from enzymatically digested tissue including cardiomyocytes, adipocytes, endocardial cells, lymphatics, epicardial cells and mast cells (Fig. 1e and Extended Data Figs. 2 and 3).

**Fig. 2 ∣:**
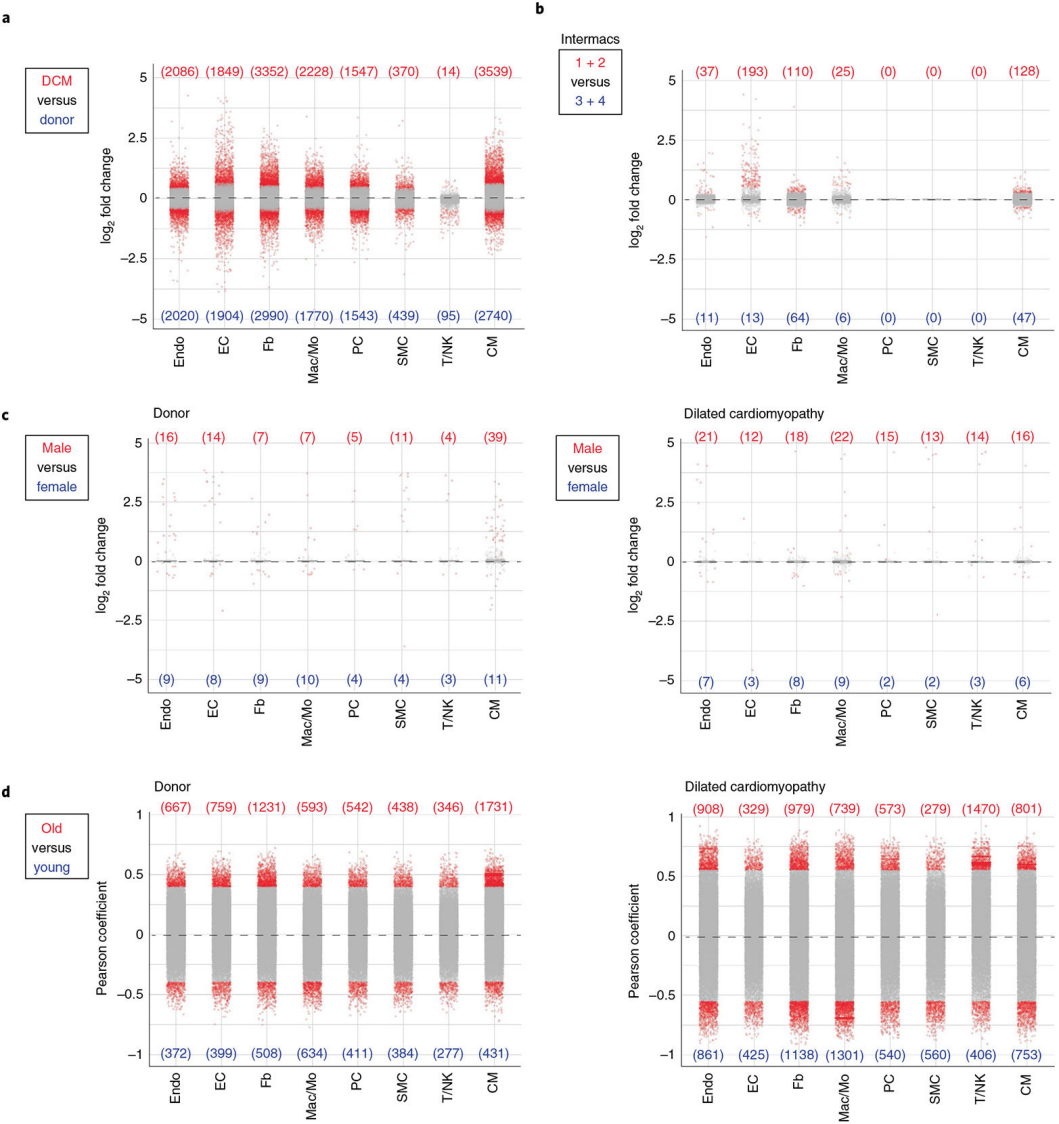
Differential influence of disease state, sex and age on cell type-specific gene expression. **a**–**c**, Dot plots showing pseudobulk (DESeq2) based differential gene expression across major cell populations. Differential expression was calculated from snRNA-seq data for disease (**a**, Donor versus DCM), INTERMACS score (**b**, 1 and 2 versus 3 and 4) and sex (**c**, male versus female) are shown. **d**, Genes correlated with age by Pearson coefficient are also shown. Genes with adjusted *P* value <0.05 are colored in red and genes with adjusted *P* value >0.05 are colored in gray (*P* value calculated using Wald test adjusted for multiple test correction). Number of upregulated and downregulated genes with adjusted *P* value <0.05 per cell type is displayed in parenthesis. Supplementary Tables 21-26 contain a complete list of genes.

The analyzed dataset was powered to investigate the influence of age, sex and disease state and severity on gene expression. Differential expression analysis using pseudobulk and single-cell approaches demonstrated substantial overlap (Supplementary Tables 4 and 5). Disease state had the most powerful influence on differential gene expression across cell types (Fig. 2a and Supplementary Table 21). Heart failure severity, as assessed by INTERMACS profile/score (predictor of outcomes in the advanced heart failure population) revealed evidence of differential expression in cardiomyocytes, endothelial, endocardial, fibroblast and myeloid cells (Fig. 2b and Supplementary Table 22)^22,23^. We also observed changes in cardiac cell composition as a function of disease state. Individuals with DCM had decreased numbers of cardiomyocytes, pericytes and mast cells; and increased numbers of fibroblasts, myeloid cells, T/NK cells and lymphatics (Supplementary Table 6). Many of these changes were observed in both men and women (Supplementary Tables 7 and 8). Heart failure severity was not associated with changes in cell composition (Supplementary Table 9).

**Extended Data Fig. 4 ∣:**
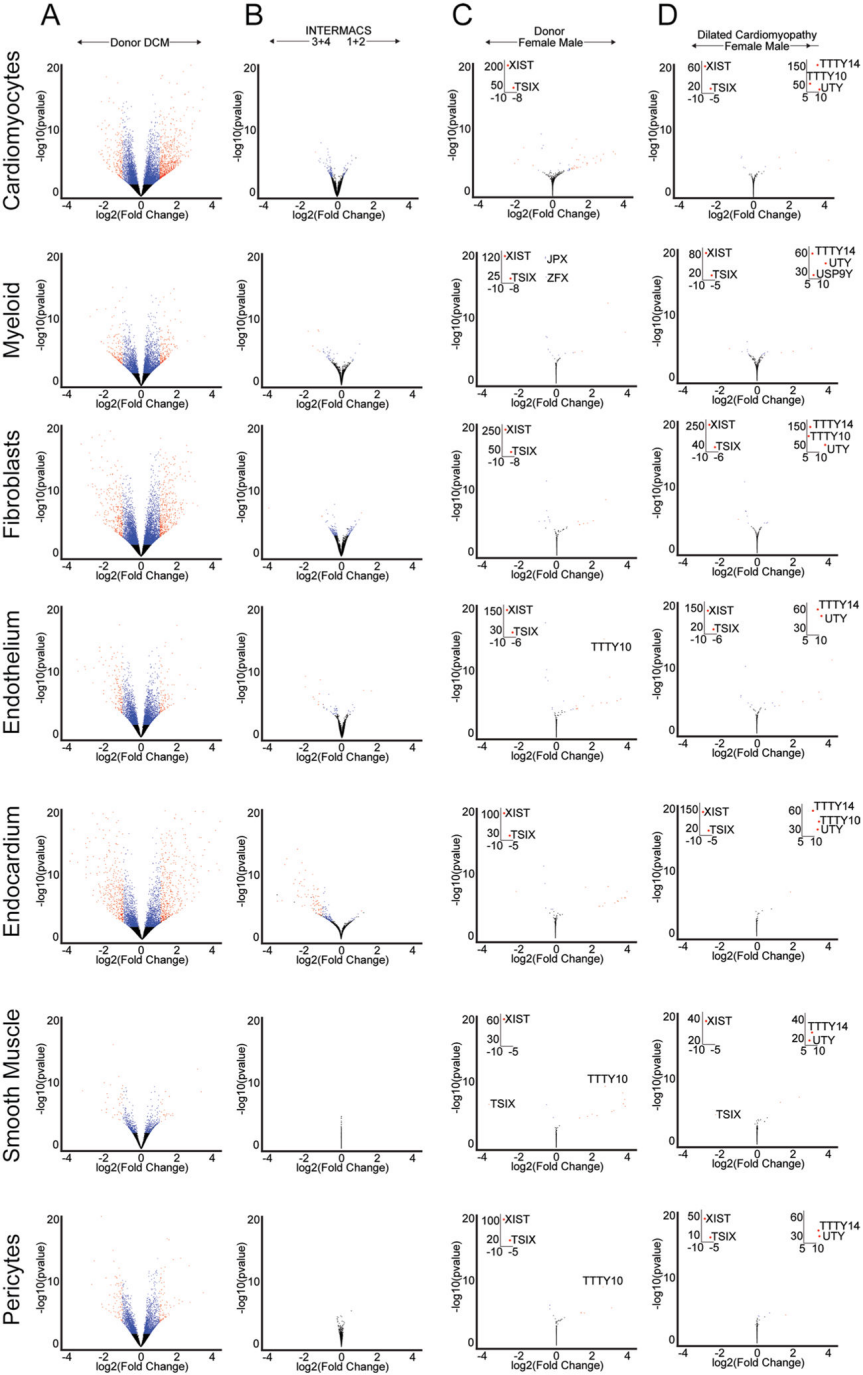
Pseudobulk differential expression reveals the contribution of disease state, sex and disease severity across major cell types. A, Volcano plots of pseudobulk differential expression analysis of single-nucleus RNA sequencing data performed on each cell type comparing donor control vs. dilated cardiomyopathy (DCM). B, Volcano plots of pseudobulk differential expression analysis of single nucleus RNA sequencing data performed on each cell type comparing disease severity (INTERMACS score 3+4 vs 1+2, lower score indicates more advance disease). C-D, Volcano plots of pseudobulk differential expression analysis of single nucleus RNA sequencing data performed on each cell type comparing sex separated by donor (C) and DCM (D). Insets represent values outside of the plotted area. See Supplementary Tables 21-26 for complete list of genes.

Substantially fewer differentially expressed genes (DEGs) were detected comparing sex in either non-diseased donors or individuals with DCM. The majority of differentially expressed transcripts were located on the X and Y chromosomes including *XIST*, *TSIX* and *TTTY* genes (Fig. 2c and Extended Data Fig. 4). We did not detect clear differences in cell composition between male and female donors or individuals with DCM (Supplementary Tables 10, 11, 23 and 24).

**Extended Data Fig. 5 ∣:**
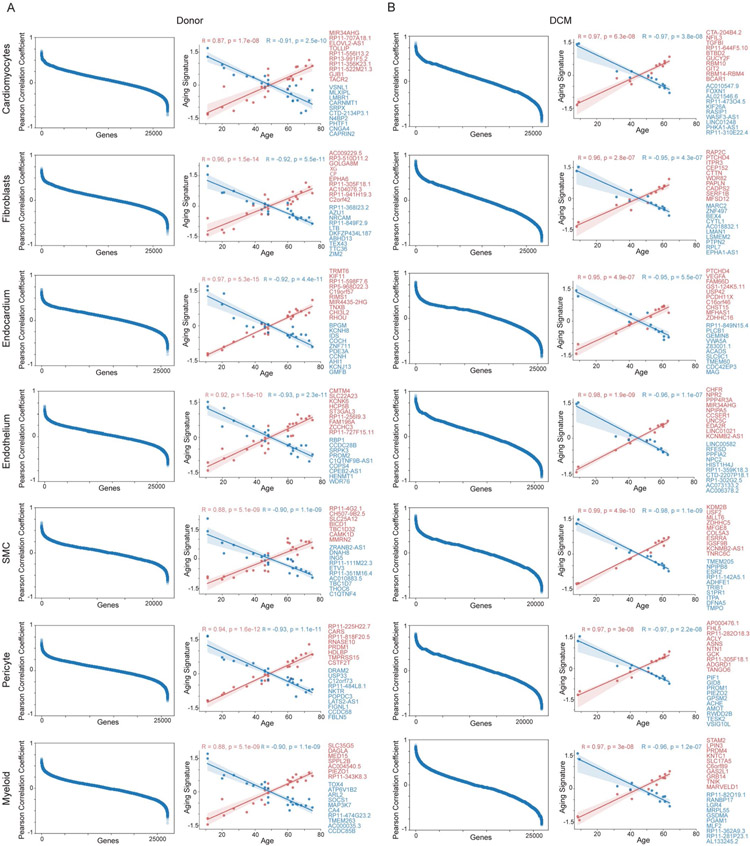
Pseudobulk differential expression reveals gene expression correlation with age in donor and diseased hearts. A-B, Plot of genes versus Pearson correlation coefficient (left) and linear regression using the top 10 genes correlated with age ranked by Pearson coefficient. Line of best fit is displayed (red-positively correlated, blue-negatively correlated, genes listed in respective colors, points represent individual samples, p-values calculated using 2-tailed linear regression Wald test with t-distribution, shaded areas represent 95% confidence interval, Donor; n=25, DCM; n=13) for donor (A) and DCM (B). Pearson Coefficients were calculated for all expressed genes from single nucleus dataset in relation to age as a continuous variable. See Supplementary Tables 25-26 for complete list of genes.

To identify changes in cardiac cell composition and gene expression associated with age, we computed positive and negative relationships using Pearson correlation. This analysis was separately performed in donor and DCM cohorts to account for the possibility that relationship between age, cell composition and gene expression may differ in the context of health and heart failure. We observed that myeloid cell number was associated with older age in donor hearts, a finding that was most evident in females. We did not observe significant age-associated alterations in major cell populations in DCM hearts (Supplementary Tables 12, 13, 25 and 26). In contrast, we identified multiple genes that were associated with younger and older age across cell types in donor and DCM hearts (Fig. 2d). We constructed age-associated gene signatures by selecting genes with Pearson correlation coefficients >0.6 or <−0.6. Regression analysis revealed robust age-associated gene signatures across cell types. Notably, age-associated gene expression signatures were cell type-specific, differed by disease state and similarly evident in male and female patients (Extended Data Fig. 5 and Supplementary Tables 14-17). We also detected distinct pathways associated with age in donor controls and individuals with DCM (Supplementary Tables 18 and 19).

Given that disease state was associated with the most robust changes in cell composition and gene expression, we chose to focus our analysis on how heart failure influences major cardiac cell populations, including cardiomyocytes, myeloid cells, fibroblasts, pericytes/smooth muscle cells, endothelial cells and endocardial cells. These populations displayed the greatest differences in gene expression (Fig. 2a).

### Cardiomyocytes phenotypically converge in dilated cardiomyopathy.

**Extended Data Fig. 6 ∣:**
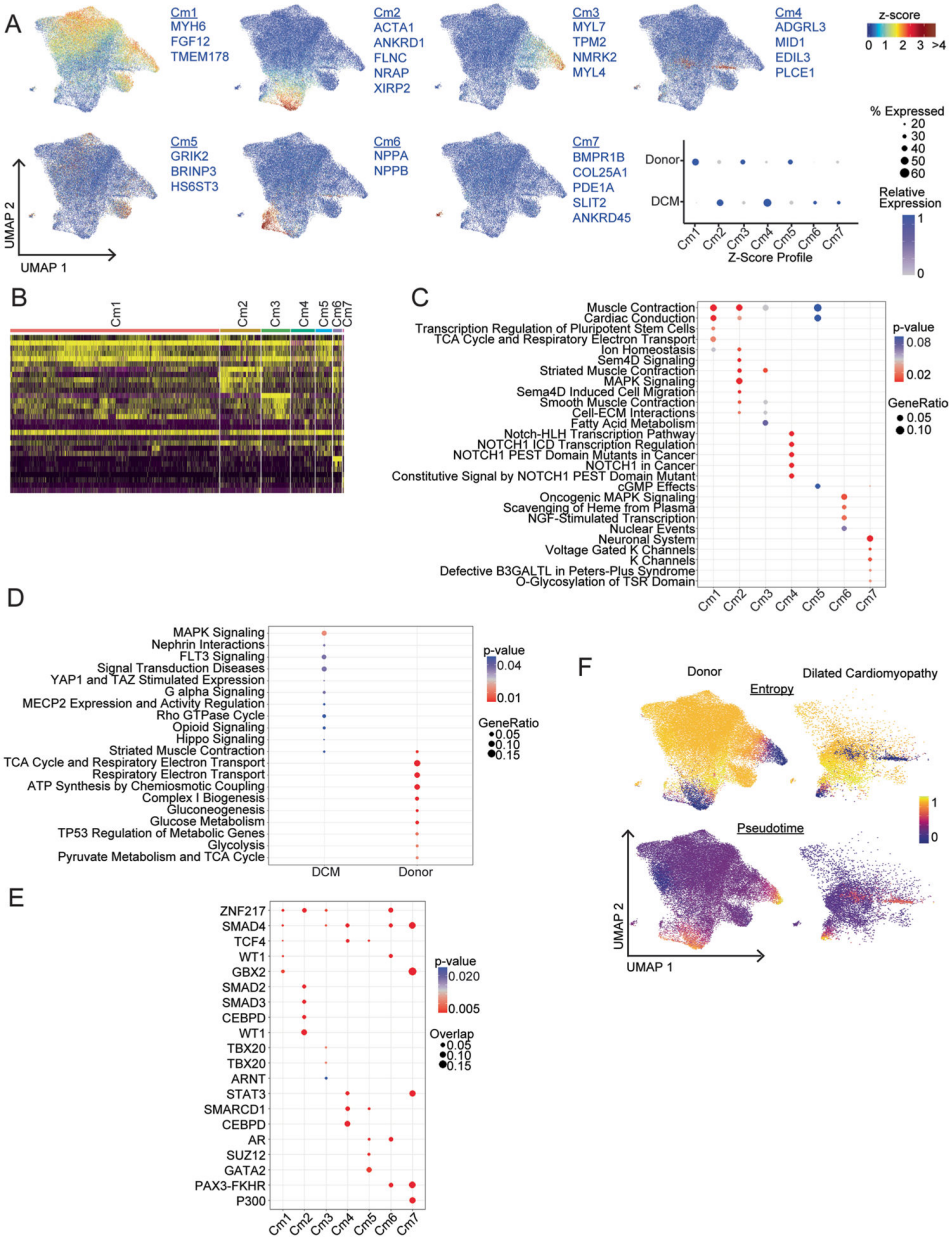
Supplement to Fig. 3 – Cardiomyocytes. A, Z-score feature plots for transcriptional signatures enriched in each cardiomyocyte state. Genes used for cell type identification (blue) were selected based on enrichment from Seurat differential expression analysis. Dot plot displays relative expression values for each Z-score split by disease state. B, Heatmap displaying top 5 enriched genes in each cell state from Seurat differential expression analysis on integrated dataset. C, enrichPathways analysis identifies pathways top differentially enriched pathways by cell state. Genes used in the analysis were selected from Seurat differential expression analyses with adjusted p<0.05. p-value calculated using hypergeometric distribution and corrected for multiple comparisons. D, enrichPathway analysis comparing enrichment of top pathways between disease states. Genes used in the analysis selected from intersection of pseudobulk and Seurat differential expression with p<0.05 and log2FC>0.1. p-value calculated using hypergeometric distribution and corrected for multiple comparisons. E, Transcription factor analysis displaying top enriched transcription factors in each cell state using ChEA 2016 database (https://maayanlab.doud/Enrichr). Genes used in the analysis selected from Seurat differential expression with p<0.05 and log2FC>0.1. p-value calculated using Fisher exact test. F, Palantir pseudotime and entropy values overlaid on UMAP projection split by disease state.

**Fig. 3 ∣:**
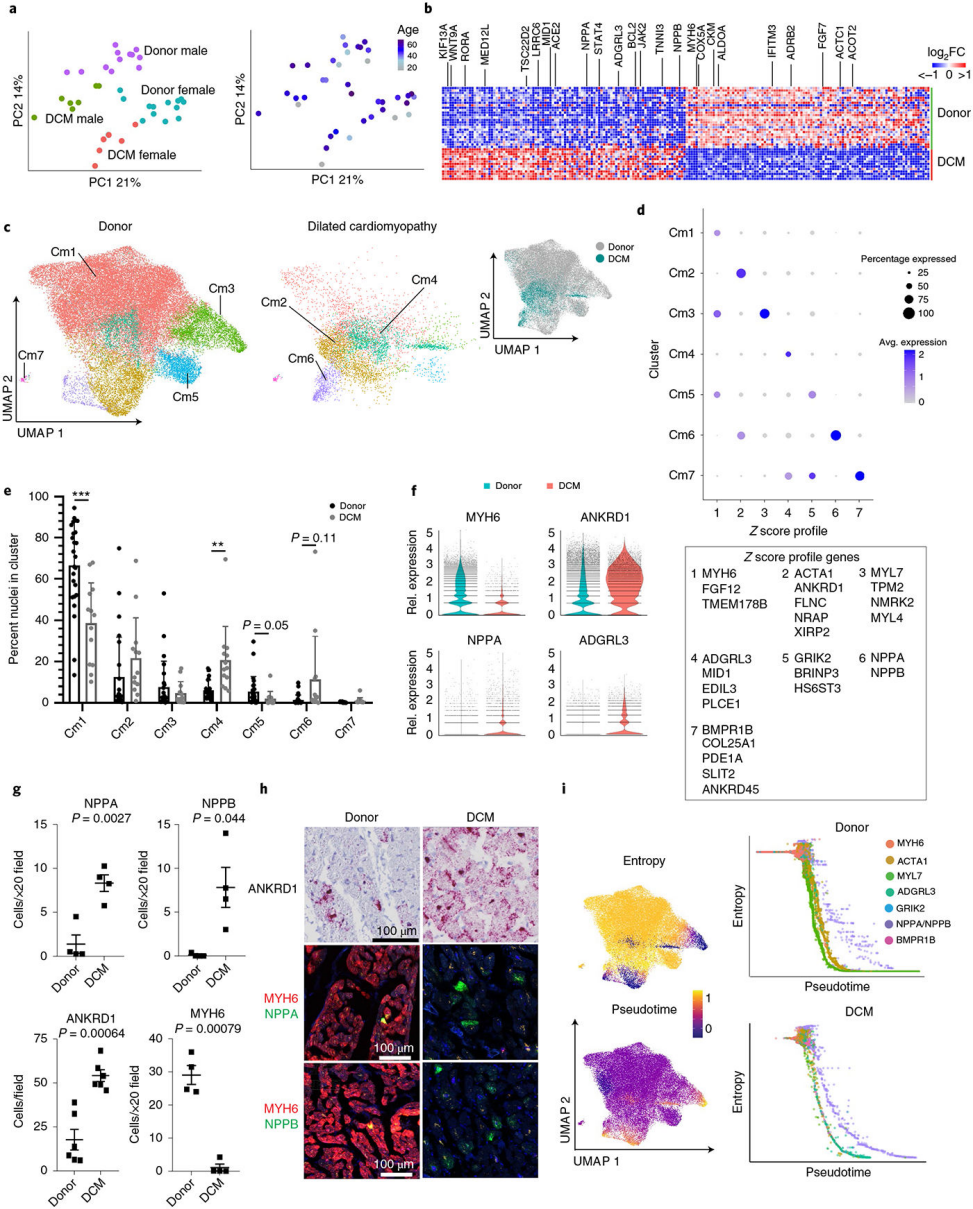
Acquisition of disease-associated cardiomyocyte states in dilated cardiomyopathy. **a**, PCA, DESeq2 plots of cardiomyocyte pseudobulk snRNA-seq data colored by sex and disease state (left) and age (right). Each data point represents an individual. **b**, Heat map displaying the top 100 upregulated and downregulated genes ranked by log_2_ fold-change comparing donor control to DCM. DEGs were derived from the intersection of pseudobulk (DESeq2) and single-cell (Seurat) analyses. **c**, Unsupervised re-clustering of donor and DCM cardiomyocytes within the integrated dataset split by disease state. Major cardiomyocyte states are labeled. Inset (right) colored by disease state demonstrates mixing within cell states. **d**, Dot plot displaying *z* scores for transcriptional signatures that distinguish cardiomyocyte states (genes selected by enrichment in Seurat differential expression analysis, listed in box below plot). **e**, Distribution of cardiomyocyte states by cluster (*P < 0.05, **P < 0.01, ***P < 0.001, Welch’s t-test, two-tailed, data represents mean ± s.d., donor; *n* = 25 samples, DCM; *n* = 13 samples). *P* values for clusters comparing donor to DCM are Cm1: 3.8 × 10^−4^; Cm2, 1.8 × 10^−1^; Cm3, 3.2 × 10^−1^; Cm4, 8.1 × 10^−3^; Cm5, 5.4 × 10^−2^; Cm6, 1.1 × 10^−1^; Cm7, 1.1 × 10^−1^. **f**, Violin plots of *MYH6*, *ANKRD1*, *NPPA* and *ADGRL3* expression in donor control and DCM cardiomyocytes. **g**, Quantification of the number of cardiomyocytes expressing *ANKRD1*, *MYH6*, *NPPA* and *NPPB* mRNA in donor control and DCM samples (*P* value from Welch’s *t*-test, two-tailed, data represents mean ± s.d. For *ANKRD1*, donor; *n* = 6 samples, DCM; *n* = 6 samples. For *MYH6*, *NPPA* and *NPPB*, donor; *n* = 4 samples, DCM; *n* = 4 samples). **h**, Representative RNA in situ hybridization images (RNAScope) of indicated genes. **i**, Palantir pseudotime trajectory analysis of cardiomyocytes showing entropy and pseudotime scores overlaid on the UMAP projection (left). Entropy versus pseudotime plots of donor and DCM cardiomyocytes identifying differing trajectories of healthy and disease-associated cardiomyocyte states (right).

Principal-component analysis (PCA) of pseudobulk data indicated that disease state and sex had the greatest influence on gene expression variance in cardiomyocytes (Fig. 3a). Overlaying age distribution onto the PCA plot did not suggest a dominant relationship with age across all cardiomyocytes, although regression analysis did identify gene expression signatures associated with age (Fig. 2d and Extended Data Fig. 5). Genes associated with age differed in donor and DCM specimens and were distinct from genes that were differentially expressed between donor versus DCM cardiomyocytes (0.1% and 3.7% overlap, respectively). Pseudobulk differential expression analysis between men and women indicated robust differences in a modest number of genes encoded on the X and Y chromosome, possibly accounting for separation observed by PCA (Extended Data Fig. 4). Differential expression analysis by pseudobulk and single-cell approaches across disease state revealed a large number of genes significantly upregulated (*NPPA*, *NPPB*, *ACE2* and *KIF13A*) and downregulated (*MYH6*, *ADRB2* and *CKM*) in DCM samples compared to non-diseased donors (Fig. 3b). Pathway analysis identified multiple differentially regulated pathways upregulated (MAPK, FLT3, HIPPO/YAP and GCPR signaling) and downregulated (metabolism) in DCM (Extended Data Fig. 6).

Unsupervised clustering identified seven cardiomyocyte states with differing gene expression signatures (Fig. 3c,d and Extended Data Fig. 6). Cardiomyocytes from donor samples existed in all seven states marked by *MYH6* (Cm1), *ACTA1* (Cm2), *MYL7* (Cm3), *ADGRL3* (Cm4), *GRIK2* (Cm5), *NPPA/NPPB* (Cm6) and *BMPR1B* (Cm7) expression. DCM samples displayed a bias toward *ADGRL3*-expressing cardiomycytes, trend toward more *NPPA/NPPB*-expressing cardiomyocytes and marked reduction in *MYH6-* and *GRIK2-*expressing cardiomyocytes (Fig. 3e). Cardiomyocyte clusters marked by *MYH6*, *MYL7* and *GRIK2* displayed stronger expression of signature genes in donor samples, whereas cardiomyocyte clusters marked by *ACTA1*, *ADGRL* and *NPPA/NPPB* displayed stronger expression of signature genes in DCM samples (Extended Data Fig. 6). In addition, we observed a global decrease in *MYH6* expression and increases in *ANKRD1*, *NPPA* and *ADGRL3* expression in DCM (Fig. 3f). To validate shifts in cardiomyocyte state and gene expression in DCM at the tissue level, we performed RNA in situ hybridization. Compared to donor controls, we observed significant increases in *NPPA*, *NPPB* and *ANKRD1-*expressing cells and significant reduction in *MYH6*-expressing cells in DCM (Fig. 3g,h).

Pathway and transcription factor enrichment analyses performed on each cardiomyocyte cell state identify pathways that distinguished cardiomyocytes states including metabolism, muscle contraction, Semaphorin, NOTCH, MAPK signaling and potassium channels. This analysis also identified transcription factors that were predicted to regulate gene expression within each of the cardiomyocyte states (Extended Data Fig. 6).

To explore the temporal relationship between cardiomyocyte states, we performed pseudotime trajectory analysis using Palantir, a Python package that employs probabilistic models to discern complex and diverse lineage relationships^24^. We calculated pseudotime and entropy values for each cardiomyocyte cluster to predict putative states of cell differentiation (Fig. 3i and Extended Data Fig. 6). We plotted entropy versus pseudotime values for each cell and superimposed cluster designations. Donor cardiomyocytes were predicted to contain two highly differentiated cell states marked by *MYL7* and *ACTA1* expression. In contrast, DCM samples displayed two distinct highly differentiated cell states marked by *ARGRL3* and *NPPA/NPPB* expression (Fig. 3i). Collectively, these observations suggest a convergence toward disease-associated cardiomyocyte phenotypes in DCM.

### Monocyte expansion and inflammatory macrophage diversification.

**Fig. 4 ∣:**
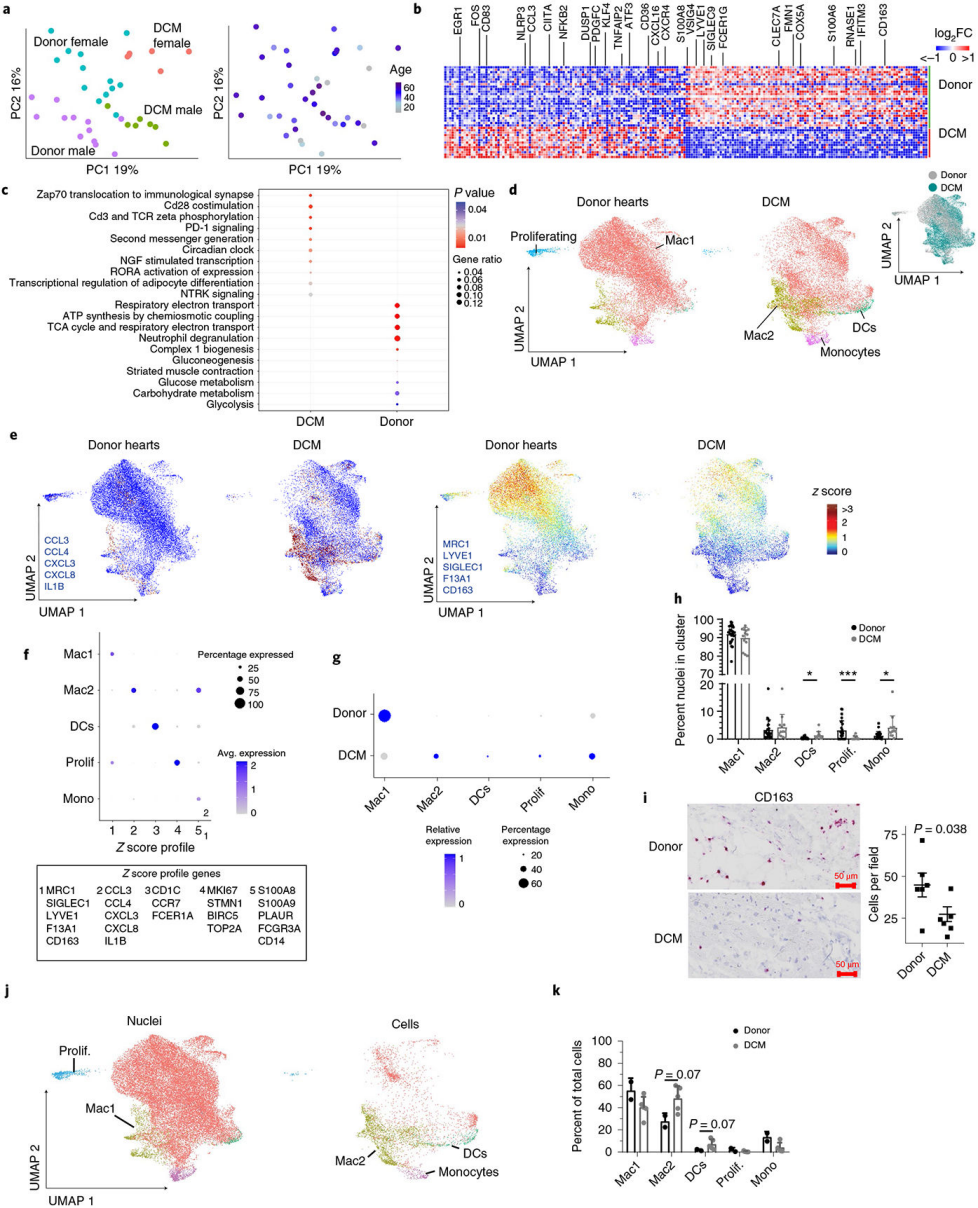
Dilated cardiomyopathy is associated with shifts in macrophage composition and gene expression favoring inflammatory populations. **a**, PCA, DESeq2 plots of monocyte, macrophage and dendritic cell pseudobulk snRNA-seq data colored by sex and disease state (left) and age (right). Each data point represents an individual. **b**, Heat map displaying the top 100 upregulated and downregulated genes ranked by log_2_ fold-change comparing donor control to DCM. DEGs were derived from the intersection of pseudobulk (DESeq2) and single-cell (Seurat) analyses. **c**, WikiPathways analysis comparing top enriched pathways in each condition. Genes were selected from the intersection of pseudobulk (DESeq2) and single-cell (Seurat) analyses with *P* < 0.05 and log_2_FC >0.1. *P* value calculated using hypergeometric distribution and corrected for multiple comparisons. **d**, UMAP of unsupervised re-clustering of monocytes, macrophages and dendritic cells within the Harmony integrated dataset split by disease state. Major cell states are labeled. Inset (right) colored by disease state demonstrates mixing within cell states. **e**, Z score feature plot of the two macrophage populations identified split by disease state (left, Mac1; right, Mac2). Genes (in blue) were selected by enrichment in the respective populations. **f**,**g**, Dot plots displaying the z scores for transcriptional signatures that distinguish monocyte, macrophage and dendritic cell populations by cell state (**f**) and split by disease state (**g**) (genes selected by enrichment in Seurat differential expression analysis are listed in box below plot). **h**, Distribution of myeloid states by cluster (*P < 0.05, ***P < 0.001, Welch’s *t*-test, two-tailed, data represents mean ± s.d., derived from single-nucleus data, donor; *n* = 25 samples, DCM; *n* = 13 samples). *P* values for clusters comparing donor to DCM are Mac1, 2.7 × 10^−1^; Mac2, 5.4 × 10^−1^; DCs, 2.6 × 10^−2^; Prolif, 9.0 × 10^−4^; Mono, 3.4 × 10^−2^. **i**, Representative RNA in situ hybridization images (RNAScope) for CD163 (red and blue, hematoxylin) and quantification of CD163^+^ cells in donor and DCM samples (*P* value from Welch’s *t*-test, two-tailed, data represents mean ± s.d., donor; *n* = 6 samples, DCM; *n* = 6 samples). CD163 is a marker of tissue-resident macrophages. **j**, UMAP plot of clusters split by sequencing technology. **k**, Distribution of myeloid states by cluster Welch’s *t*-test, two-tailed, data represents mean ± s.d., derived from only single-cell data, donor; *n* = 2 samples, DCM; *n* = 5 samples). *P* values for clusters comparing donor to DCM are Mac1, 2.1 × 10^−1^; Mac2, 6.6 × 10^−2^; DCs, 6.6 × 10^−2^; Prolif, 5.1 × 10^−1^; Mono, 1.5 × 10^−1^.

Macrophages, monocytes and dendritic cells are increasingly studied in mouse models of cardiac injury and heart failure^25-29^. We identified large populations of macrophages, monocytes and dendritic cells in donors and individuals with DCM (Fig. 1c,e). PCA of pseudobulk data indicated that disease state and sex had the greatest effect on gene variance in this population (Fig. 4a). Differential expression analysis by pseudobulk and single-cell approaches across disease state revealed a large number of genes significantly upregulated (*CCL3*, *NLRP3*, *NFKB2* and *EGR1*) and downregulated (*VSIG4*, LYVE1, *FMN1* and *CD163*) in DCM samples compared to non-diseased donors (Fig. 4b). Similar to cardiomyocytes, pseudobulk differential expression analysis between males and females indicated robust differences in a small number of genes encoded on the X and Y chromosomes, including *XIST*, *JPX* and *TTTY10* (Extended Data Fig. 4). Pathway analysis identified upregulation of multiple pathways in DCM samples including T-cell co-stimulation, PD-1 and NGF signaling, whereas metabolism pathways were downregulated in DCM (Fig. 4c).

**Extended Data Fig. 7 ∣:**
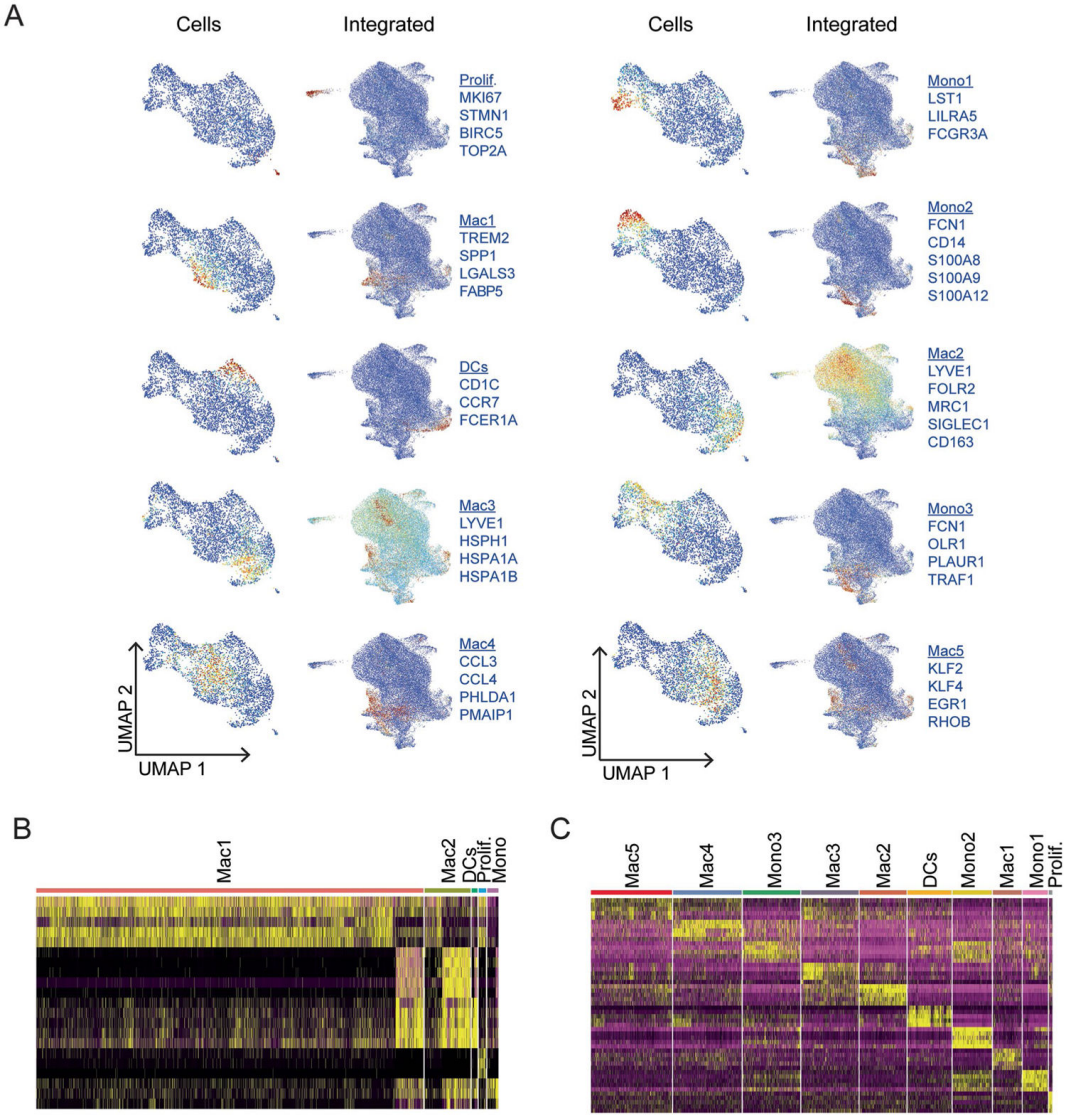
Supplement to Figs. 4 and 5 – Monocytes, macrophages and dendritic cells. A, Z-score feature plots for transcriptional signatures enriched in each monocytes, macrophages, and dendritic cells state. Genes used for cell type identification (blue) were selected based on enrichment from Seurat differential expression analysis from single cell dataset (from Fig. 5). Z-scores are overlaid on the single cell RNA sequencing (left) and integrated UMAP (right) projections. B, Heatmap displaying top 5 enriched genes in each cell state from Seurat differential expression analysis on integrated dataset. C, Heatmap displaying top 5 enriched genes in each cell state from Seurat differential expression analysis on single cell dataset.

Unsupervised clustering of the integrated dataset revealed the presence of large numbers of macrophages and smaller populations of monocytes, dendritic and proliferating cells. We identified two populations of macrophages, including a subset that expressed tissue-resident markers (Mac1: *MRC1*, *SIGLEC1*, *CD163*, *LYVE1* and *F13A1*)^30-32^ and a subset that expressed chemokines and cytokines (Mac2: *CCL3*, *CCL4*, *CXCL3*, *CXCL8* and *IL1β*). Compared to donor controls, we observed a reduction in proliferating macrophages and expansion of monocytes and dendritic cells in individuals with DCM. We also observed a reduction in the tissue-resident macrophage signature and increase in the inflammatory macrophage signature in DCM (Fig. 4d-h and Extended Data Fig. 7). RNA in situ hybridization confirmed reduction in CD163^+^ cells in DCM samples compared to donor controls (Fig. 4i).

Visualization of snRNA-seq and scRNA-seq data within the integrated object indicated a bias in recovered cell populations. While each dataset contained all of the identified cell types, the scRNA-seq dataset displayed a bias toward monocytes, dendritic cells and non-resident macrophages. The snRNA-seq dataset contained a substantially larger number of resident macrophages (Fig. 4j). To further evaluate the diversity of monocytes, dendritic cells and non-resident macrophages, we chose to focus on the scRNA-seq data. Analysis of cell composition using cluster annotations defined within the integrated dataset demonstrated increased dendritic cells and Mac2 (chemokine/cytokine-expressing) and reduced Mac1 (tissue-resident signature) in DCM (Fig. 4k).

**Fig. 5 ∣:**
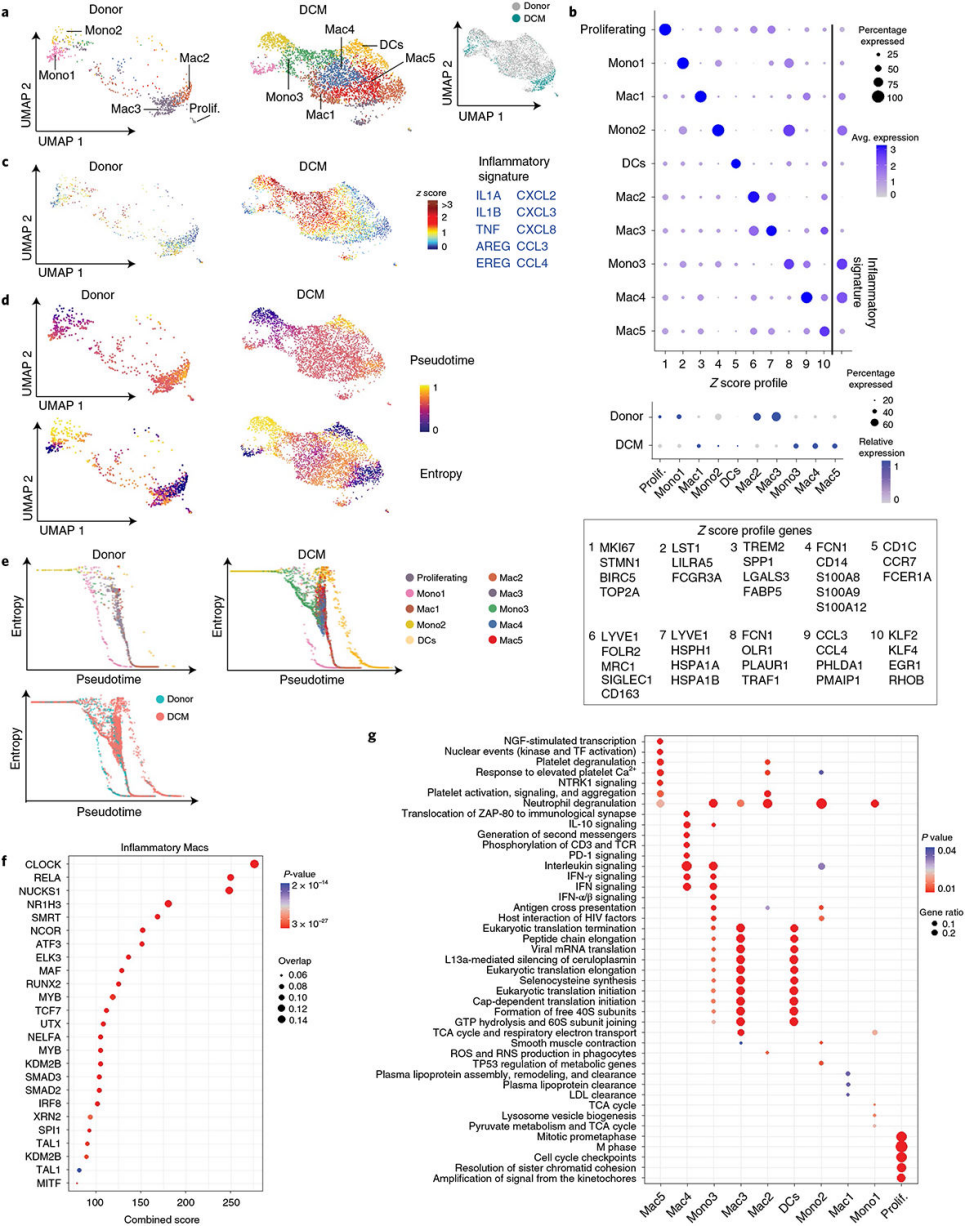
Dilated cardiomyopathy is associated with the emergence of inflammatory monocyte-derived populations. **a**, UMAP projection of unsupervised re-clustering of myeloid cells from the scRNA-seq dataset. Major cell states are labeled. Inset (right) colored by disease state demonstrates mixing within cell states. **b**, Dot plot displaying the *z* scores for transcriptional signatures that distinguish each monocyte, macrophage and dendritic cell state by cell state (above) and disease state (below) (genes selected by enrichment in Seurat differential expression analysis, listed in box below plot). **c**, *Z* score feature plot overlaying an inflammatory gene expression signature (genes in blue) on the scRNA-seq UMAP projection split by disease state. **d**,**e**, Palantir pseudotime trajectory analysis of myeloid scRNA-seq data. Entropy and pseudotime overlayed on UMAP projection split by disease state (**d**) and entropy versus pseudotime plots split by disease state identify major cell trajectories (nonclassical monocytes, resident macrophages and dendritic cells). Inflammatory cell states that emerge in DCM have high entropy and low pseudotime values, suggesting an intermediate state of differentiation. **f**, Transcription factor analysis for genes upregulated in inflammatory macrophage states (Mac1, Mac4 and Mac5) using ChEA 2016 database (https://maayanlab.cloud/Enrichr). Genes used in the analysis selected from Seurat differential expression with *P* < 0.05 and log_2_FC>0.1. *P* value calculated using Fisher’s exact test. **g**, enrichPathway analysis displaying the top five enriched pathways in each cell state. Genes used in the analysis selected from Seurat differential expression with *P* < 0.05 and log_2_FC > 0.1. *P* value calculated using hypergeometric distribution and corrected for multiple comparisons.

Unsupervised clustering revealed the presence of discrete monocytes (Mono1, nonclassical-*FCGR3A*; Mono2, classical-*CD14*; Mono3, intermediate-*OLR1*), macrophages (Mac1, *TREM2*; Mac2, *FOLR2/LYVE1*; Mac3, *LYVE1/HSPH1;* Mac4, *CCL3*; Mac5, *KLF2*) and dendritic cells (*CD1C*) (Fig. 5a,b). We observed shifts in monocyte, macrophage and dendritic cell composition between donor and DCM groups. Donor samples contained classical and nonclassical monocytes as well as two populations of LYVE1^+^ resident macrophages (Mac2 and Mac3). DCM samples displayed reduced numbers of resident macrophages and a greater number of intermediate monocytes, dendritic cells and three additional macrophage populations (Mac1, Mac4 and Mac5). Classical and intermediate monocytes and macrophages marked by *CCL3*, *TREM2* and *KLF2* expressed robust levels of inflammatory mediators including *IL1A*, *IL1B*, *TNF*, *AREG* and *EREG* and multiple chemokines (Fig. 5c).

To infer the differentiation state of monocyte, dendritic cell and macrophage populations, we utilized Palantir. Calculation of pseudotime and entropy values demonstrated that CD14^+^ monocytes (Mono2) represented the most progenitor-like state. CD16^+^ monocytes (Mono1), dendritic cells and resident macrophages (Mac2 and Mac3) represented the most differentiated cells, each with distinct trajectories. Compared to donors, we observed an accumulation of cells with intermediate differentiation states along the macrophage trajectory in DCM samples. Superimposing cluster identities revealed that these cells belonged to the intermediate monocyte (Mono3), *TREM2* (Mac1), *CCL3* (Mac4) and *KLF2* (Mac5) clusters, suggesting that they are monocyte-derived (Fig. 5d,e). Transcription factor analysis identified enrichment for targets of transcription factors, including *CLOCK*, *RELA*, *MYB*, *RUNX2*, SMAD2/3 and *IRF8* in the inflammatory macrophage states (Mac1, Mac4 and Mac5; Fig. 5f). Comparison of pathways across cell states identified enrichment of unique pathways in individual states included pathways involved in inflammation, interferon and interleukin signaling (Fig. 5g). These data provide a link between monocyte-derived macrophages and inflammation in DCM.

### Fibroblasts diversify in dilated cardiomyopathy.

**Extended Data Fig. 8 ∣:**
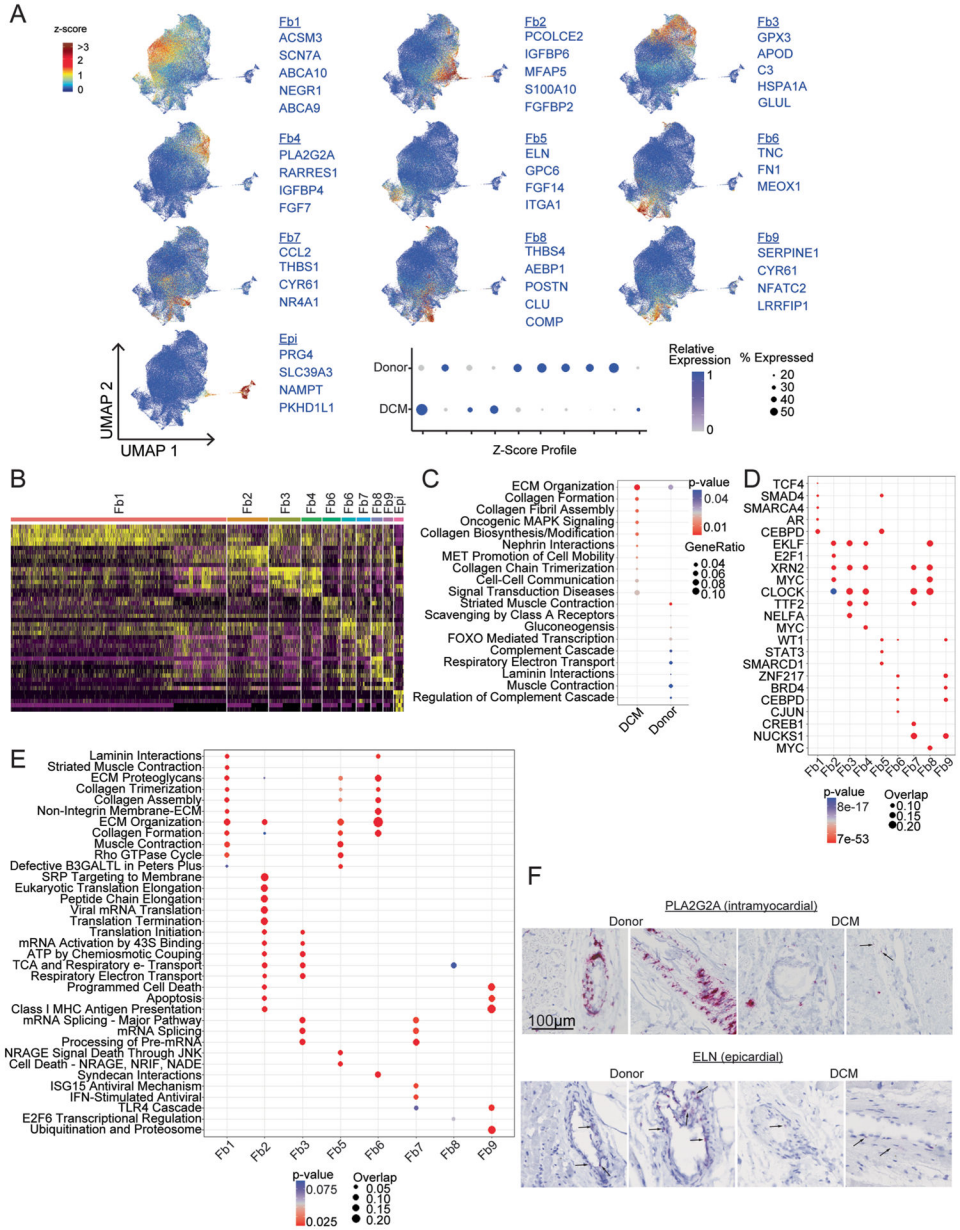
Supplement to Fig. 6 – Fibroblasts. A, Z-score feature plots for transcriptional signatures enriched in each fibroblast state. Genes used for cell type identification (blue) were selected based on enrichment from Seurat differential expression analysis. Z-scores are overlaid on the integrated UMAP projections. Dot plot displays relative expression values for each Z-score split by disease state. B, Heatmap displaying top 5 enriched genes in each cell state from Seurat differential expression analysis on integrated dataset. C, WikiPathways analysis identifies pathways differentially enriched by disease state. Genes used in the analysis included the intersection of pseudobulk and Seurat differential expression analyses with adjusted p<0.05. p-value calculated using hypergeometric distribution and corrected for multiple comparisons. D, Transcription factor analysis displaying top enriched transcription factors in each cell state using ChEA 2016 database (https://maayanlab.cloud/Enrichr). Genes used in the analysis selected from Seurat differential expression with p<0.05 and log2FC>0.1. p-value calculated using Fisher exact test. E, enrichPathway analysis comparing enrichment of pathways between cell states. Genes used in the analysis selected from Seurat differential expression with p<0.05 and log2FC>0.1. p-value calculated using hypergeometric distribution and corrected for multiple comparisons. F, RNA in situ hybridization for PLA2G2A and ELN (red). Representative images showing perivascular staining of PLA2G2A in the myocardium of donor samples. Minimal staining was observed in DCM samples. ELN staining was observed in the media of epicardial coronary arteries in both donor and DCM samples.

**Fig. 6 ∣:**
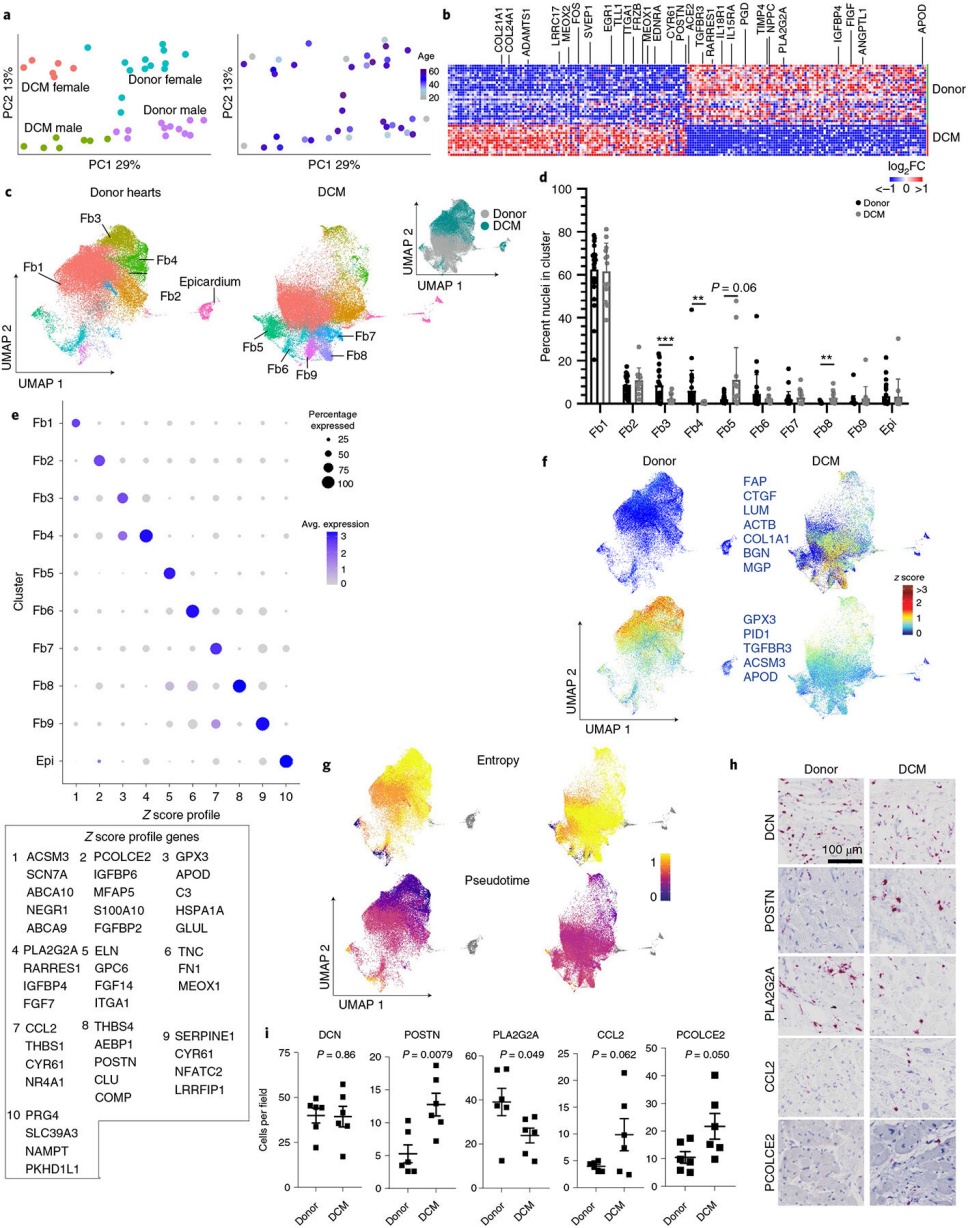
Phenotypic shifts and emergence of disease-associated fibroblasts in dilated cardiomyopathy. **a**, PCA, DESeq2 plots of fibroblast pseudobulk snRNA-seq data colored by sex and disease state (left) and age (right). Each data point represents an individual. **b**, Heat map displaying the top 100 upregulated and downregulated genes ranked by log_2_ fold-change comparing donor control to DCM. DEGs were derived from the intersection of pseudobulk (DESeq2) and single-cell (Seurat) analyses. **c**, Unsupervised re-clustering of donor and DCM fibroblasts and epicardium within the integrated dataset split by disease state. Major fibroblast states are labeled. Inset (right) colored by disease state demonstrates mixing within cell states. **d**, Distribution of fibroblast states by cluster (*P < 0.05, **P < 0.01, ***P < 0.01, Welch’s *t*-test, two-tailed, data represents mean ± s.d., donor; *n* = 25 samples, DCM; *n* = 13 samples). *P* values for clusters comparing donor to DCM are Fb1, 8.3 × 10^−1^; Fb2, 3.0 × 10^−1^; Fb3, 4.2 × 10^−4^; Fb4, 5.1 × 10^−3^, Fb5; 5.9 × 10^−2^; Fb6, 2.6 × 10^−1^; Fb7, 5.3 × 10^−1^; Fb8, 7.5 × 10^−3^; Fb9, 4.0 × 10^−1^; Epi, 9.1 × 10^−1^. **e**, Dot plot displaying the *z* scores for transcriptional signatures that distinguish fibroblast states (genes selected by enrichment in Seurat differential expression analysis, listed in box below plot). **f**, *Z* score feature plot of transcriptional signatures associated with DCM (top) and with donor (bottom) fibroblast states. Plot is split by disease state. DCM fibroblasts are enriched in genes associated with activation. Enriched genes (blue) were defined using Seurat differential gene expression analysis. **g**, Palantir pseudotime trajectory analysis of integrated fibroblast RNA-seq data. Entropy and pseudotime overlayed on UMAP projection split by disease state. **h**, Representative RNA in situ hybridization images (RNAScope) of indicated genes (red) counterstained with hematoxylin (blue). **i**, Quantification of the number of cells expressing *DCN*, *POSTN*, *PLA2G2A*, *CCL2* and *PCOLCE2* mRNA in donor control and DCM samples (*P* value from Welch’s *t*-test, two-tailed, data represent mean ± s.d., donor; *n* = 6 samples, DCM; *n* = 6 samples).

We identified a large population of cardiac fibroblasts in donor controls and DCM hearts. PCA demonstrated that variability across fibroblast samples was driven by disease state and sex (Fig. 6a). Differences between males and females were driven by a small number of genes encoded on the X and Y chromosomes, including *XIST*, *JPX* and *ZFYAS1* (Extended Data Fig. 4). Pseudobulk and single-cell differential expression analysis identified a large number of genes that were significantly upregulated (*POSTN*, *MEOX1/2*, *TLL1*, *EDNRA*, *SVEP1* and *FRZB*) and downregulated (*APOD*, *NPPC*, *ANGPTL1*, *FIGF* and *ACE2*) in DCM samples compared to non-diseased donors. Pathway analysis identified upregulated (extracellular matrix synthesis and organization, MAPK and nephrin signaling) and downregulated (metabolism, biosynthesis, complement and muscle contraction) pathways in DCM (Extended Data Fig. 8).

Unsupervised clustering of the integrated dataset revealed multiple distinct populations of fibroblasts (Fig. 6c). The majority of fibroblasts in both donor and DCM hearts displayed a conserved gene expression signature characteristic of fibroblasts (Fb1, Fb2). We identified two fibroblast subpopulations primarily present in donor controls that expressed *GPX3* (Fb3) and *PLA2G2A* (Fb4), respectively. We observed additional minor fibroblast subpopulations characterized by the expression of *ELN* (Fb5), *TNC* (Fb6), *CCL2* (Fb7), *THBS4* (Fb8) and *SERPINE1* (Fb9). Epicardial cells were also represented (Epi). *POSTN*, a marker of disease-associated fibroblasts was expressed in Fb8 ^33^. Fb5 and Fb8 were found at increased abundance in DCM (Fig. 6c-e and Extended Data Fig. 8). Fibroblasts in DCM hearts displayed a robust activation signature that included *FAP*, *CTGF*, *LUM*, *ACTB*, *COL1A1*, *BGN* and *MGP* expression. Donor fibroblasts selectively expressed a signature represented by *GPX3*, *PID1*, *TGFBR3*, *ACSM3* and *APOD* (Fig. 6f). Palantir identified fibroblasts marked by *ELN*, *TNC* and *SERPINE1* expression as the most differentiated cell states based on low entropy and high pseudotime values. All other fibroblasts seemed to exist in a state of high entropy, suggesting substantial plasticity within these populations (Fig. 6g). Pathway analysis comparing fibroblast states identified distinct pathway enrichment, including pathways involved in extracellular matrix synthesis and assembly, protein translation, messenger RNA processing, cell death, type I interferon signaling, TLR4 signaling, metabolism and the ubiquitin–proteome system. Transcription factor analysis identified enrichment of targets of specific transcription factors in the majority of fibroblast cell states (Extended Data Fig. 8).

We validated shifts in fibroblast composition between donor controls and DCM hearts using RNA in situ hybridization for select fibroblast populations. The overall numbers of fibroblasts (marked by *DCN* expression) remained similar between donor control and DCM hearts. Notably, we observed that fibroblast subpopulations were located either within the interstitial space between cardiomyocytes (*PCOLCE2*-Fb2, *CCL2*-Fb7 and *POSTN*-Fb8), adjacent to distal vasculature (*PLA2G2A*-Fb4) or surrounding epicardial coronary arteries (*ELN*-Fb5). The number of *POSTN CCL2*, and *PCOLCE2*-expressing fibroblasts was increased in DCM samples. *PLA2G2A*-expressing fibroblasts were increased in donor hearts (Fig. 6h,i and Extended Data Fig. 8).

### Pericytes and smooth muscle cells.

**Extended Data Fig. 9 ∣:**
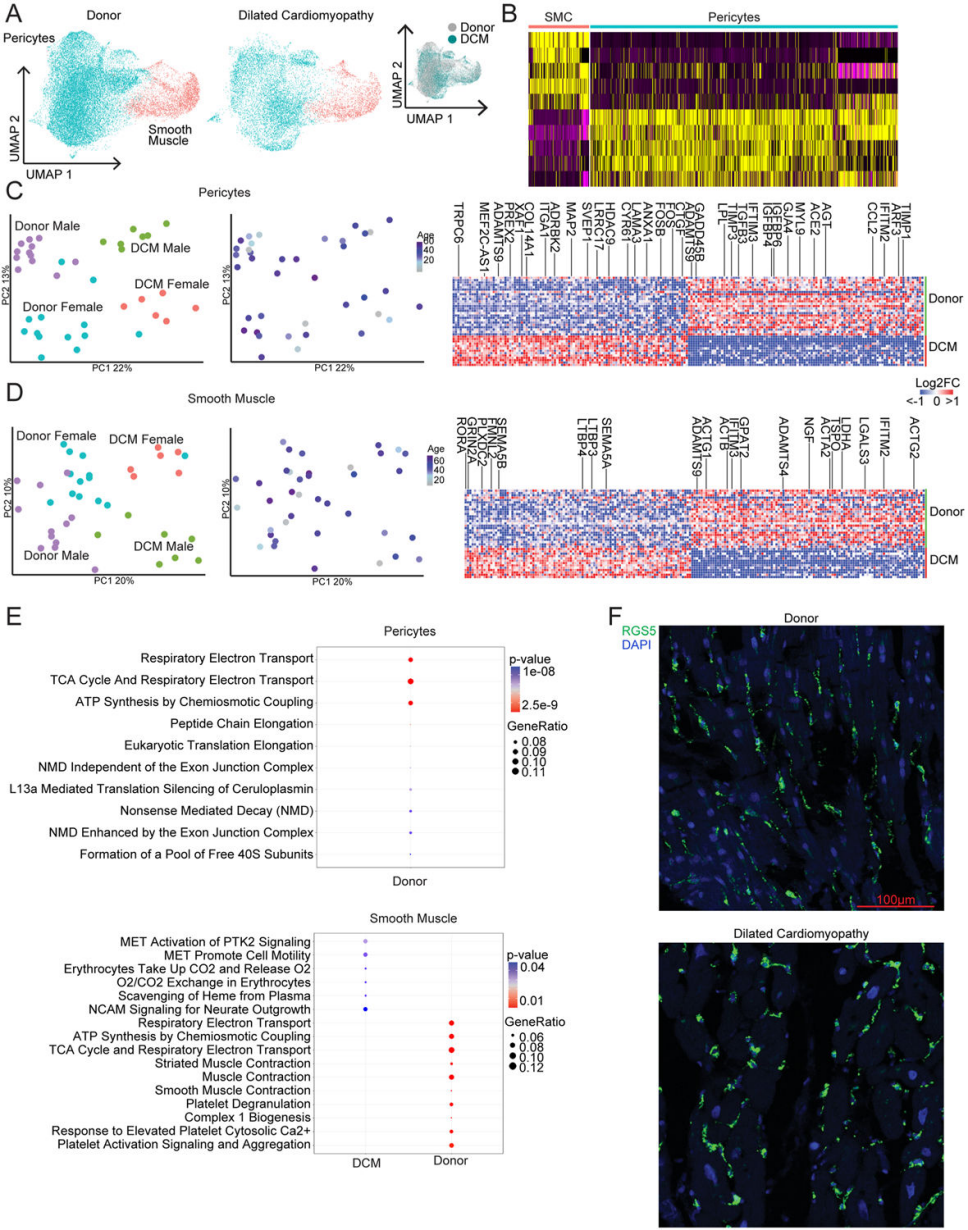
Pericytes and smooth muscle cells exhibit global changes in gene expression in dilated cardiomyopathy. A, Unsupervised clustering of pericytes and fibroblasts within the integrated dataset split by disease state. Inset panel (right) colored by disease state demonstrates mixing within cell states. B, Heatmap displaying top 5 enriched genes in each cell population from Seurat differential expression analysis on integrated dataset. C-D, Principal-component analysis (PCA, DESeq2) plots of pericyte (C) and smooth muscle cell (D) pseudobulk single nucleus RNA sequencing data colored by sex and disease state and age. Each data point represents an individual subject. Heatmaps displaying the top 100 upregulated and downregulated genes ranked by log2 fold-change comparing donor control to dilated cardiomyopathy (DCM). Differentially expressed genes were derived from the intersection of pseudobulk (DESeq2) and single cell (Seurat) analyses. E, WikiPathways analysis identifies top differentially enriched pathways in pericytes (top) and smooth muscle cells (bottom) by disease state. No pathway enrichment was detected in DCM pericytes. Genes used in the analysis included the intersection of pseudobulk and Seurat differential expression analyses with adjusted p<0.05 and log2FC>0.1. p-value calculated using hypergeometric distribution and corrected for multiple comparisons. F, Representative images of RGS5 staining for pericytes by RNA in situ hybridization.

Unsupervised clustering of pericytes and smooth muscle cells revealed minimal heterogeneity within each of these populations. PCA demonstrated that variability across samples was driven by disease state and sex. Differences between males and females were driven by a small number of genes encoded on the X and Y chromosomes (Extended Data Fig. 4). Pseudobulk and single-cell differential expression and pathway analyses identified genes and pathways enriched in DCM pericytes and smooth muscle cells compared to non-diseased donors. Within pericytes, *TRPC6*, *ITGA1*, *XAF1*, *CYR61* and *CTGF* were upregulated in DCM and *TIMP1*, *CCL2*, *AGT*, *ACE2*, *IFITM2/3* and *TGFB3* were downregulated in DCM. Among smooth muscle cells, *RORA*, *PLXNDC2*, LTBP3/4 and *SEMA5A* were upregulated in DCM and *ACTG1/2*, *ACTB*, *LGALS3*, *LDHA*, *IFITM2/3* and *NGF* were downregulated in DCM (Extended Data Fig. 9).

### Endothelial cells display shifts in global gene expression.

**Fig. 7 ∣:**
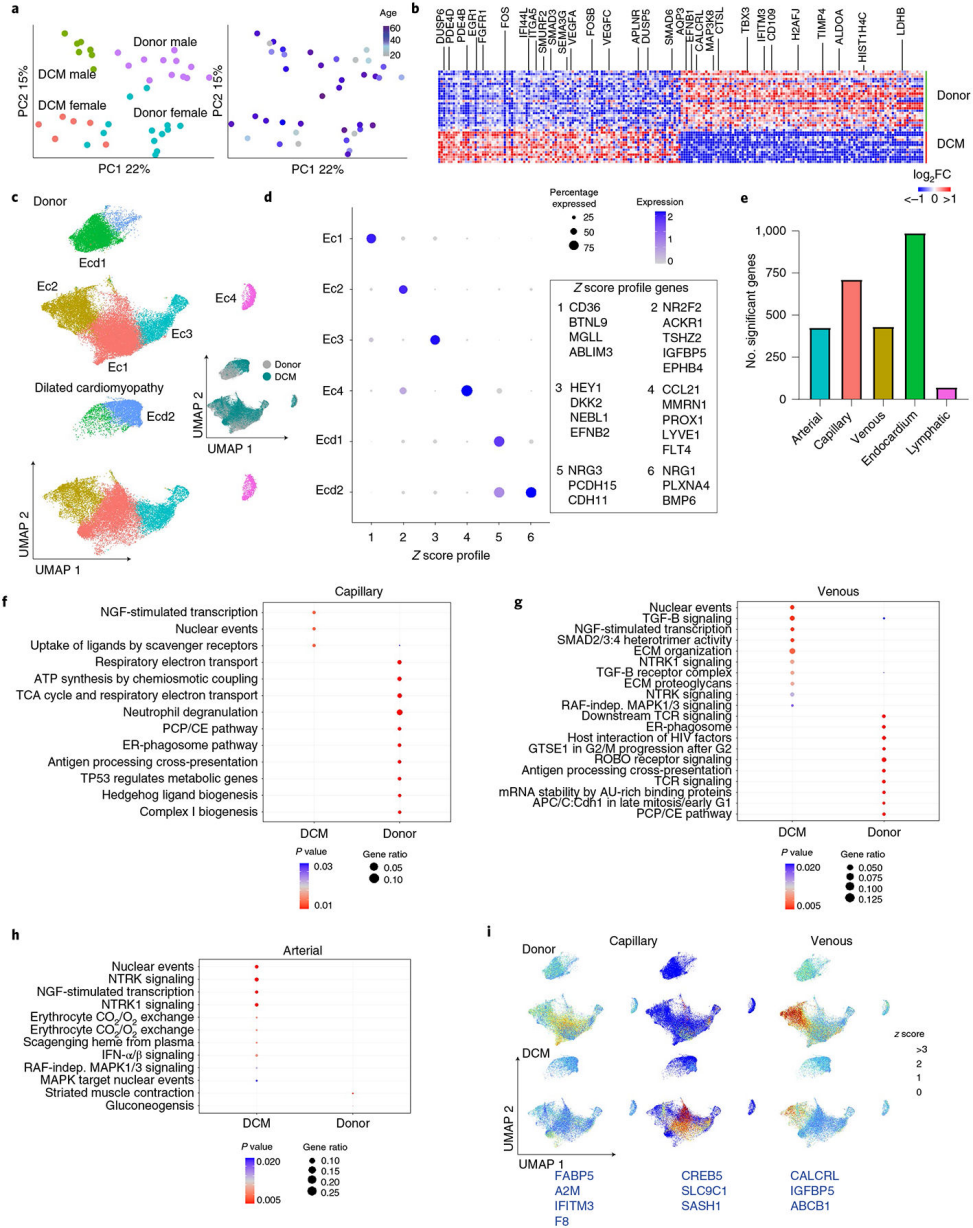
Endothelial cells exhibit global gene expression shifts in dilated cardiomyopathy. **a**, PCA, DESeq2 plots of vascular endothelial cell pseudobulk snRNA-seq data colored by sex and disease state (left) and age (right). Each data point represents an individual. **b**, Heat map displaying the top 100 upregulated and downregulated genes ranked by log_2_ fold-change comparing donor control to DCM. DEGs were derived from the intersection of pseudobulk (DESeq2) and single-cell (Seurat) analyses. **c**, Unsupervised re-clustering of donor and DCM endothelial and endocardial cells within the integrated dataset split by disease state. Major endothelial states are labeled. Inset (right) colored by disease state demonstrates mixing within cell states. **d**, Dot plot displaying z scores for transcriptional signatures that distinguish endothelial cell populations (genes selected by enrichment in Seurat differential expression analysis, genes listed in the box to right of plot). **e**, Bar graph of the number of DEGs per endothelial population (intersection of DESeq2 and Seurat differential expression analyses with adjusted *P* < 0.05 (Wilcoxon rank-sum), log_2_FC > 0.1). **f**, WikiPathways analysis identifying top differentially enriched pathways in donor and DCM capillary endothelial cells. Genes used in the analysis selected from intersection of pseudobulk and Seurat differential expression with *P* < 0.05 and log_2_FC > 0.1. *P* value calculated using hypergeometric distribution and corrected for multiple comparisons. **g**, WikiPathways analysis identifying top differentially enriched pathways in donor and DCM venous endothelial cells. Genes used in the analysis selected from intersection of pseudobulk and Seurat differential expression with *P* < 0.05 and log_2_FC > 0.1. *P* value calculated using hypergeometric distribution and corrected for multiple comparisons. **h**, WikiPathways analysis identifying top differentially enriched pathways in donor and DCM arterial endothelial cells. Genes used in the analysis selected from intersection of pseudobulk and Seurat differential expression with *P* < 0.05 and log_2_FC > 0.1. *P* value calculated using hypergeometric distribution and corrected for multiple comparisons. **i**, *Z* score feature plots of transcriptional signatures associated with donor and DCM groups in capillary and venous endothelial cells split by disease state. Genes (blue) were selected by enrichment in the differential expression analyses.

Endothelial cells within the heart include arterial, venous, capillary, lymphatic and endocardial cells. PCA of artery, vein and capillary pseudobulk data identified disease state and sex as the most distinguishing features (Fig. 7a). Differences between males and females were driven by a small number of genes encoded on the X and Y chromosomes (Extended Data Fig. 4). Pseudobulk and single-cell differential expression analysis in vascular endothelial cells (arteries, veins, capillaries) identified a large number of genes significantly upregulated (*DUSP5/6*, *PDE4B/D*, *EGR1*, *FGFR1*, *SMAD3/6*, *VEGF-A/C* and *APLNR*) and downregulated (*LDHB*, *ALDOA*, *IFITM3*, *TBX3* and *AQP3*) in DCM samples compared to donors (Fig. 7b).

**Extended Data Fig. 10 ∣:**
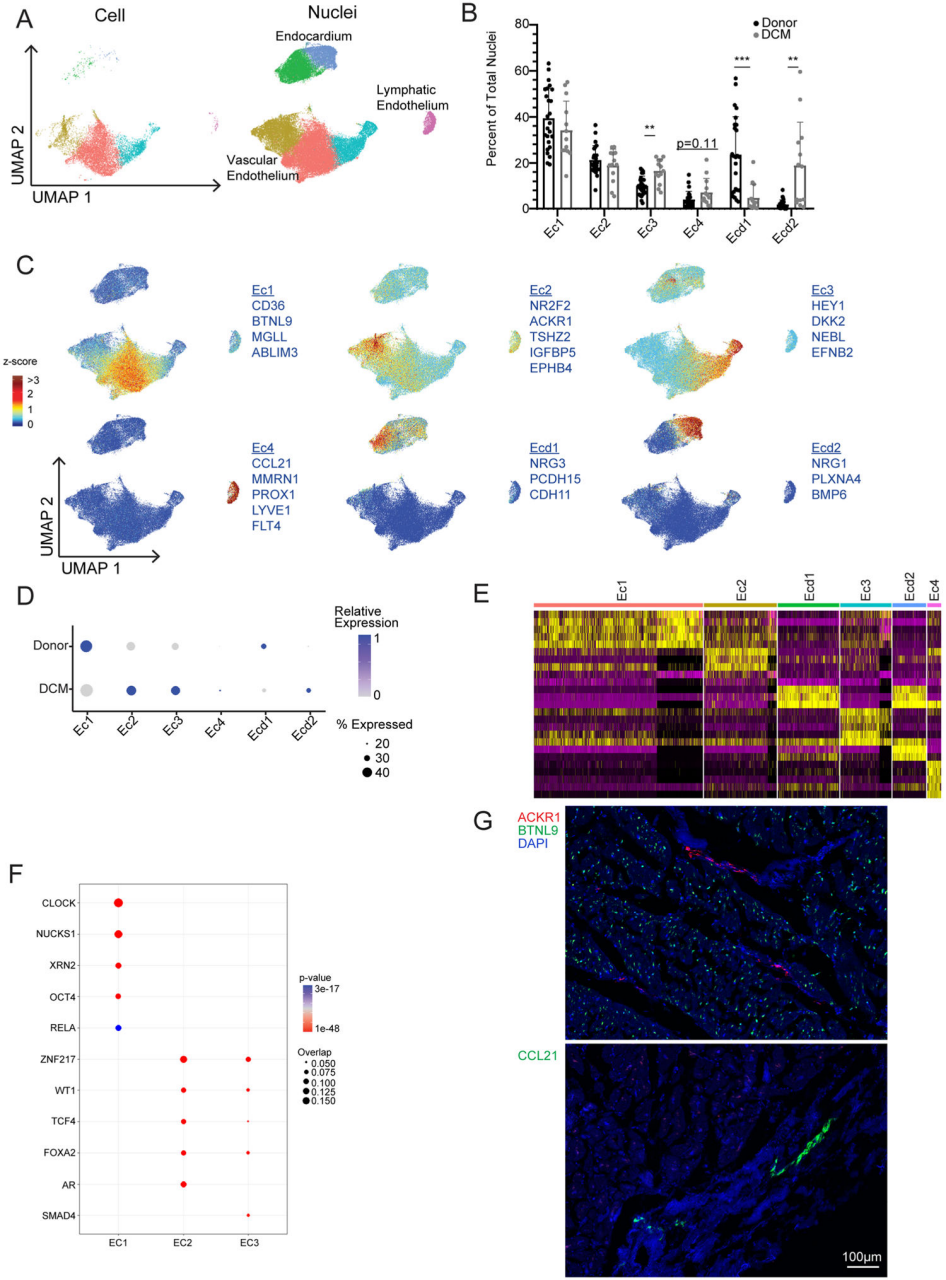
Supplement to Fig. 7 – Endothelial cells. **A**, UMAP projection of the integrated dataset split by technology (single cell vs. single nucleus RNA sequencing) and colored by subpopulation. **B**, Distribution of nuclei in the integrated object divided by major cell type (*<0.05, **<0.01, ***<0.001 by Welch’s T-test, two-tailed, data represents mean ± standard deviation, Donor; n=25 samples, DCM; n=13 samples). p-values for clusters comparing Donor to DCM are; Ec1:1.9e-1, Ec2: 3.0e-1, Ec3: 1.5e-3, Ec4: 1.1e-1, Ecd1: 3.3e-5, Ecd2: 6.7e-3. **C**, Z-score feature plots for transcriptional signatures enriched in endothelial and endocardial cell populations. Genes used for cell type identification (blue) were selected based on enrichment from Seurat differential expression analysis. Z-scores are overlaid on the integrated dataset. **D**, Dot plot of relative expression values for each Z-score split by disease state. **E**, Heatmap displaying top 5 enriched genes in each cell state from Seurat differential expression analysis on integrated dataset. **F**. Transcription factor analysis displaying top enriched transcription factors in each cell state using ChEA 2016 database (https://maayanlab.cloud/Enrichr). p-value calculated using Fisher exact test. Genes used in the analysis selected from Seurat differential expression with p<0.05 and log2FC>0.1. **G**, Representative RNAScope images of vascular (top) and lymphatic (bottom) endothelial cells. *ACKR1* – venous, *BTNL9* – capillary, *CCL21* – lymphatic.

Vascular endothelial cells and endocardial cells displayed distinct transcriptional signatures and clustered separately (Fig. 7c,d and Extended Data Fig. 10). Within the integrated object, the snRNA-seq dataset contained all major endothelial cell populations, whereas the scRNA-seq dataset displayed a bias toward arterial (Ec3), venous (Ec2) and capillary (Ec1) endothelial cells. Few endocardial (Ecd1 and Ecd2) or lymphatic (Ec4) cells were recovered from scRNA-seq data (Extended Data Fig. 10). Quantification of endothelial cell populations revealed an increase in arterial endothelial cells (Ec3) and a shift in endocardial cell state in DCM (Extended Data Fig. 10). We did not observe further diversification of arterial, venous, capillary, lymphatic or endocardial cells. Instead, we observed global shifts in gene expression between control and DCM samples (Fig. 7c,d and Extended Data Fig. 10). Utilizing RNA in situ hybridization, we visualized expression of recognized venous (*ACKR1*), capillary (*BTNL9*) and lymphatic (*CCL21*) markers identified from Seurat differential expression analysis (Extended Data Fig. 10)^34,35^.

Pseudobulk and single-cell differential gene expression analysis of snRNA-seq data revealed that endocardial cells and capillaries displayed the greatest number of DEGs between donor control and DCM conditions. Arterial and venous endothelial cells displayed a modest number of DEGs and lymphatics had few differentially expressed genes (Fig. 7e). Among vascular endothelial cells, capillaries displayed enrichment for pathways associated with NGF signaling in DCM and metabolism, ER-phagosomes and hedgehog signaling in donor controls. Venous endothelial cells displayed enrichment for pathways involved in TGF-β, NGF, NTRK1 and MAPK signaling in DCM and mitosis, ER-phagosome, planar cell polarity and ROBO signaling in donor controls. Arterial endothelial cells displayed enrichment for pathways involved in NGF, NTRK1, type I interferon and MAPK signaling in DCM and gluconeogenesis and muscle contraction in donor controls (Fig. 7f-h). We also identified cell-specific signatures associated with disease state. *FABP5*, *A2M*, *IFITM3* and *F8* expression was enriched in donor capillaries, whereas *CREB5*, *SLC9C1* and *SASH1* expression was enriched in DCM capillaries. Donor venous cells selectively expressed a signature represented by *CALCRL*, *IGFBP5* and *ABCB1* expression (Fig. 7i).

**Fig. 8 ∣:**
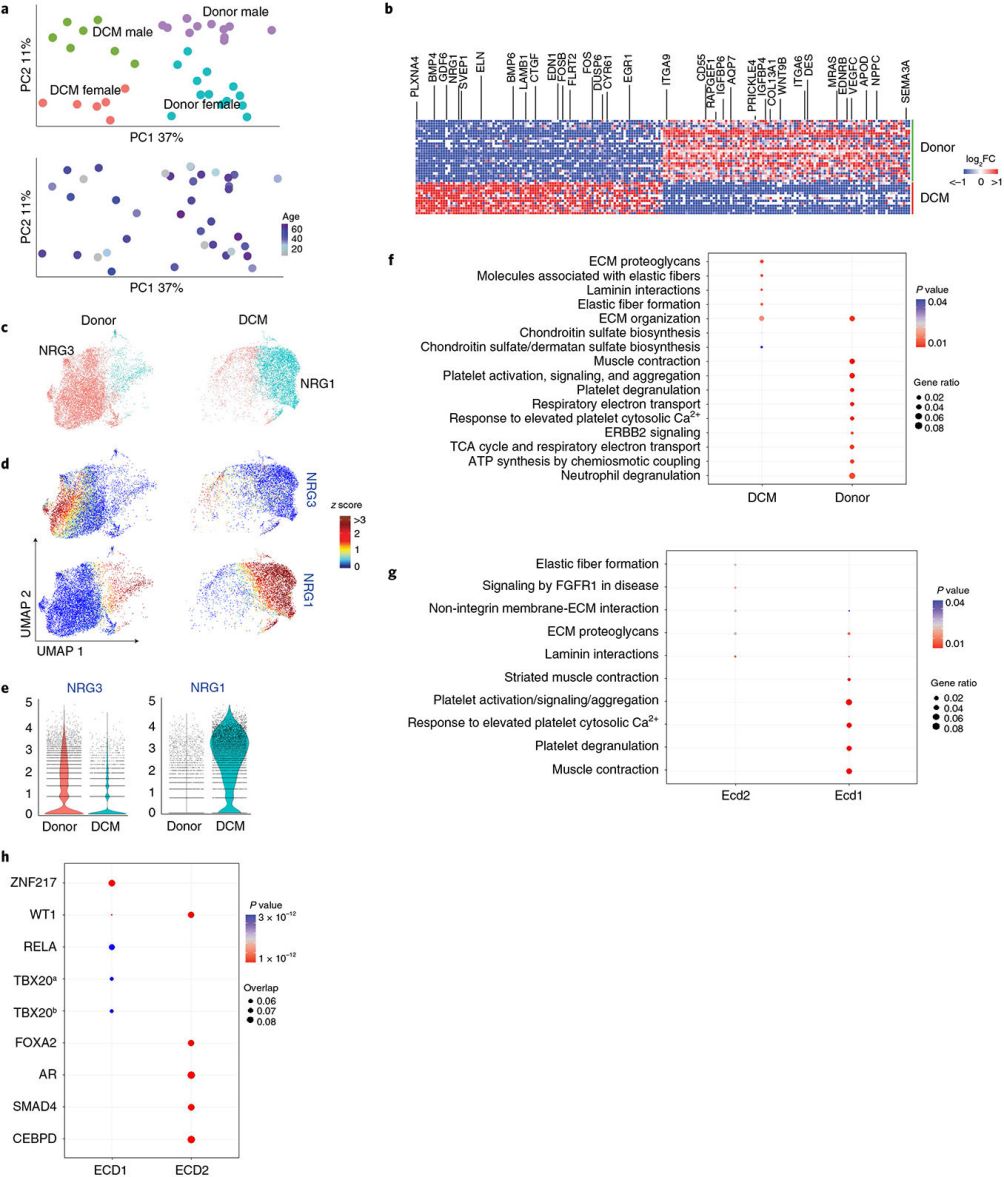
Endocardial cells exhibit distinct gene signatures in dilated cardiomyopathy. **a**, PCA, DESeq2 plots of endocardial cell pseudobulk snRNA-seq data colored by sex and disease state and age. Each data point represents an individual. **b**, Heat map displaying the top 100 upregulated and downregulated genes ranked by log_2_FC comparing donor control to DCM. DEGs were derived from the intersection of pseudobulk (DESeq2) and single-cell (Seurat) analyses. **c**, Unsupervised re-clustering of donor and DCM endocardial cells split by disease state. **d**, UMAP feature plots of *NRG*1 and *NRG3* split by disease state. **e**, Violin plots displaying *NRG1* and *NRG3* expression in endocardial cells from donor and DCM samples. **f**, WikiPathways analysis identifying top differentially enriched pathways in donor and DCM endocardial cells. Genes used in the analysis selected from intersection of pseudobulk and Seurat differential expression with *P* < 0.05 and log_2_FC > 0.1. *P* value was calculated using hypergeometric distribution and corrected for multiple comparisons. **g**, WikiPathways analysis identifying top differentially enriched pathways in endocardial cell states. Genes used in the analysis selected from Seurat differential expression with *P* < 0.05 and log_2_FC > 0.1. *P* value calculated using hypergeometric distribution and corrected for multiple comparisons. **h**, Transcription factor analysis displaying top enriched transcription factors in each cell state using the ChEA 2016 database (https://maayanlab.cloud/Enrichr). Genes used in the analysis selected from Seurat differential expression with *P* < 0.05 and log_2_FC > 0.1. *P* value calculated using Fisher’s exact test. TBX20^a^ and TBX20^b^ represent enrichment identified from two independent CHIP-seq experiments (ChEA_term 22080862, 22328084).

Similar to other populations, PCA of endocardial pseudobulk data identified disease state and sex as the most distinguishing features (Fig. 8a). Within endocardial cells, we observed a large number of genes to be significantly upregulated (*BMP4/6*, *GDF6*, *NRG1*, *SVEP1*, *ELN*, *CTGF*, *EDN1* and *CYR61*) and downregulated (*SEMA3A*, *NPPC*, *EDNRB*, *VEGF-C*, *WNT9B*, *IGFBP4/6*, *CD55* and *ITGA6/9*) in DCM samples compared to non-diseased donors (Fig. 8b). Endocardial cells were independently clustered across disease state (Fig. 8c). Donor endocardial cells (Edc1) expressed NRG3. Endocardial cells from DCM samples (Edc2) displayed strong upregulation of NRG1 and reduced NRG3 expression (Fig. 8d,e). Pathway analysis identified enrichment of pathways associated with extracellular matrix components and organization in DCM (Ecd2) and platelet activation, ERBB2 signaling, FGFR1 signaling, metabolism and muscle contraction in donor controls (Ecd1; Fig. 8f,g). We also identified enrichment for targets of transcription factors, including *FOXA2*, *AR*, *SMAD4* and *CEBPD* in Ecd2 (*NRG1* endocardial cells) and *ZNF217*, *WT1*, *TBX20* and *RELA* in Ecd1 (*NRG3* endocardial cells; Fig. 8h).

## Discussion

Single-cell technologies offer powerful new tools to dissect cell types that reside within healthy and diseased tissues. Recently, these approaches were leveraged to provide a deeper understanding of the cellular composition of the healthy human heart^10,11^. While considerable interest exists, only limited data are available to decipher how the cellular and transcriptional landscape of the heart is impacted by disease^12,13^. Using an approach that integrated snRNA-seq and scRNA-seq data from 45 individuals encompassing 220,752 nuclei and 49,723 cells, we identified 15 major cardiac cell types, uncovered cell type-specific transcriptional programs, revealed age and disease-associated gene expression signatures and observed the emergence of cell states associated with heart failure.

Aging is associated with a decline in cardiac function and subsequent adverse clinical outcomes, including heart failure. Very little is known regarding how individual cardiac cell types transcriptionally change as an individual ages. We leveraged pseudobulk methods to dissect age-associated cell type-specific gene signatures in donor and DCM hearts. The pseudobulk approach allowed us to focus on patient-level data and minimize noise inherent at the single-cell scale.

We did not observe profound associations between cellular composition and age. Only myeloid cells were found to be increased with age in donor hearts; however, we did uncover specific transcriptional signatures across most cell types that were associated with age and differed between donor and DCM hearts. For example, *TOLLIP* expression correlated with increasing age in donor cardiomyocytes, consistent with mouse data that *Tollip* expression correlates with aging and structural cardiac changes in older mice^36^. *TGFBI* and *NFIL3* positively correlated with aging in DCM cardiomyocytes. Previous studies have implicated *Nfil3* in the gene regulatory network involved in cardiac senescence and aging and *Tgfbi* as an upstream regulator of mTOR activation in *Drosophila* models of aging and cardiac disease^37,38^. Notably, *TGFBI* and *NFIL3* expression positively correlated with age only in DCM and not donor cardiomyocytes. We also identified age-related changes in genes associated with mechanical sensing in pericytes and myeloid cells. *PIEZO1* expression positively correlated with age in donors but not those with DCM. Previous reports have implicated PIEZO1 activation as an upstream signal to trigger TRPV4 channel opening, which we recently showed regulates activation of resident cardiac macrophages and cardiac adaptive remodeling^39-41^. Together, these findings highlight the presence of cell type- and disease state-specific transcriptional networks modulating aging.

We did not detect marked differences in cellular composition related to sex. However, we did detect genes that were robustly increased in men and women across cell types. Many of the identified genes (*XIST*, *JPX*, *ZFYAS1*, *TTTY10* and *TSIX*) are encoded on the X and Y chromosomes. This observation is consistent with a recent publication indicating that sex chromosomes control transcriptional and proteomic differences between male and female hearts that arise before gonad formation in mice^42^. While we did not identify sex-dependent effects on cell-type-specific gene expression in the contexts of aging and disease state, we cannot exclude the possibility that sex may have effects that were not readily identified in our analysis.

With respect to disease state, we observed robust changes in gene expression across nearly all myocardial cell types and considerable variation in how different cardiac cell populations responded to heart failure. Cardiomyocytes converged toward common disease-associated cell states, whereas fibroblasts and myeloid cells underwent dramatic diversification including the acquisition of disease-specific phenotypes. In contrast, endothelial cells, endocardial cells and pericytes displayed global transcriptional shifts without changes in cell complexity.

Previous studies examining differences across cardiac chambers have identified evidence of cardiomyocyte heterogeneity in the healthy human heart^10,11^. We identified multiple transcriptionally distinct cardiomyocyte states within the LV of non-diseased donors and individuals with DCM. Donor hearts contained seven cardiomyocyte states marked by *MYH6*, *MYL7*, *GRIK2*, *NPPA/NPPB*, *ADGRL3*, *ACTA1* and *BMPR1B* expression. DCM cardiomyocytes uniformly expressed high levels of *ANKRD1*, contained fewer *MYH6* or *GRIK2*-expressing cardiomyocytes and instead, were enriched for states identified by *ADGRL3* and *NPPA/NPPB* expression. NPPA and NPPB expression are known to identify diseased cardiomyocytes in humans^13^. Notably, *ANKRD1* expression was recently found to be enriched in cardiomyocytes from patients with adolescent versus pediatric DCM and increased in cardiomyocytes from mouse hearts that fail to regenerate^12,14^. Pseudotime trajectory analysis identified three highly differentiated cardiomyocyte states (*MYL7*, *ACTA1* and *NPPA/NPPB*) in donor hearts. In contrast, we observed two highly differentiated cardiomyocyte states in DCM marked by *ADGRL3* and *NPPA/NPPB* expression. These observations suggest that cardiomyocytes converge toward a common disease-associated state in DCM. Further understanding of the instructive cues and parental cardiomyocyte populations that give rise to *ADGRL3* and *NPPA/NPPB*-expressing cardiomyocytes may provide new insights and opportunities to intervene in the pathogenesis of human heart failure.

We observed notable transcriptional changes in non-cardiomyocyte populations (fibroblasts, macrophages, endothelial cells and endocardial cells) between healthy controls and DCM samples. Previous snRNA-seq studies have reported astounding diversity among fibroblasts in the healthy human heart^10,11,43,44^. Fibroblasts are known to expand in heart failure and acquire an activated phenotype characterized by the expression of fibroblast activated protein (*FAP*) and periostin (*POSTN*)^33,45-50^. While previous single-cell studies have identified cardiac fibroblast subsets in the healthy human heart, little is known regarding how these populations are influenced by disease. We identify multiple distinct fibroblast populations in both healthy and diseased samples with differing transcriptional signatures and spatial distribution, including elastin (*ELN*)-expressing macrophages located within the medium of coronary arteries. Fibroblasts marked by *POSTN*, *CCL2* and *PCOLCE2* were enriched in DCM, whereas *GPX3-* and *PLA2G2A*-expressing fibroblasts were enriched in donor controls. In addition, we identified an activation signature that included *FAP*, *CTGF*, *LUM*, *ACTB*, *COL1A1*, *BGN* and *MGP* that was selectively expressed in fibroblasts from DCM hearts. Differential expression analysis comparing donor and DCM fibroblasts identified upregulation of *POSTN*, *MEOX1/2*, *TLL1*, *EDNRA* and *FRZB* in DCM. *Meox1* is a homeodomain-containing transcription factor that regulates fibroblast activation in the mouse heart following stress. *Meox1* directly binds to and activates the *Postn* promotor in mice^51^. Elimination of FAP-expressing fibroblasts is sufficient to ameliorate myocardial fibrosis in mice^52^. *TLL1* regulates mature collagen formation and is linked to coronary artery disease^53^. Endothelin and Wnt signaling are known regulators of fibrosis^54,55^. These findings provide further evidence that phenotypic shifts in fibroblasts are a hallmark of heart failure.

Heterogeneity of myeloid populations, including macrophages, is increasingly appreciated to contribute to the variety of cardiac pathologies including heart failure^19,56-60^. The majority of these studies have focused on mouse models with only targeted validation in human specimens^20,27,28,61^. Consistent with small animal models, we observe a variety of monocyte, macrophage and dendritic cell populations within the human heart. The abundance of macrophages expressing a tissue-resident signature is reduced in DCM, a finding evident in mouse models of cardiac injury^19,59^. The number of proliferating macrophages was reduced in DCM, consistent with the concept that self-replication may be a trait of tissue-resident macrophages. We also observed an emergence of monocyte and macrophage populations expressing inflammatory mediators in the failing heart. Cell trajectory analysis predicted that many of these inflammatory populations represented intermediate states derived from CD14^+^ monocytes. Indeed, inhibition of monocyte recruitment or administration of anti-inflammatory agents is sufficient to reduce cardiac inflammation and myocardial fibrosis^18,59,62,63^. Future studies are needed to draw causal links and define signaling mechanisms by which inflammatory populations of macrophages regulate fibroblast activation.

While scRNA-seq and snRNA-seq provided sufficient resolution to identify major perivascular populations (arteries, veins, capillaries, pericytes, smooth muscle cells, lymphatics and endocardial cells), we did not observe additional diversity within these populations; however, we did uncover global shifts in gene expression within each of these populations between control and DCM specimens. Previous studies have identified similar shifts in global endothelial cell expression but were unable to parse contributions from each major endothelial cell type^13^. Endocardial cells displayed robust numbers of DEGs between control and DCM specimens. NRG1 and NRG3 were exclusively expressed in DCM and control endocardial cells, respectively. Notably, mouse studies identified that cardiomyocyte specific loss of NRG3 receptors (ErbB2 and ErbB4) results in spontaneous heart failure suggesting a potential role for NRG3 in regulating cardiac homeostasis^64-67^.

snRNA-seq captured cell types that are difficult to recover from enzymatically digested tissue, including cardiomyocytes, adipocytes, mast cells, epicardium, endocardium and lymphatics. Using data integration and reference mapping, we were able to effectively combine snRNA-seq and scRNA-seq data and identify at least 15 major cardiac cell populations. Current scRNA-seq datasets exploring human heart failure are small and lack the sample size necessary to elucidate the impact of disease on common and rare cardiac cell types^12,13^. scRNA-seq data provided greater depth at the expense of biased cell recovery. For example, within myeloid cells, scRNA-seq data was biased toward monocytes and intermediate macrophage populations with fewer resident macrophages recovered. These datasets were leveraged to provide additional granularity into monocytes and inflammatory macrophage populations.

This study is not without limitations. We categorized patients with DCM into a single cohort based on the lack of underlying coronary artery disease. It is likely that the exact etiology of DCM contributes to shifts in cell diversity and transcriptional state. Our dataset includes only transcriptomic information. Addition of cell-surface protein expression and chromatin accessibility information may offer additional resolution. In conclusion, this study represents a large analysis of the cellular and transcriptomic landscape of the healthy and failing human heart. We provide valuable insights into how cardiac cell populations change during heart failure including the emergence of disease-specific cell states. These data provide a valuable resource that will open up new areas of investigation and opportunities for therapeutic development and innovation.

## Methods

### Statement on human specimens.

This study complies with all relevant ethical regulations and was approved by the Washington University Institutional Review Board (study no. 201104172). All samples were procured and informed consent obtained by Washington University School of Medicine. No compensation was provided for participation. Biospecimen Reporting for Improved Study Quality data including distribution of sex, age and race can be found in Supplementary Tables 1 and 20.

### Sample preparation for scRNA-seq.

Fresh cardiac tissues from LVAD cores or identical regions from the apex of explanted donors were minced with a razor blade and transferred into a 15-ml conical tube containing DMEM with Collagenase I (450 U ml^−1^), DNase I (60 U ml^−1^) and hyaluronidase (60 U ml^−1^) and incubated at 37 °C for 1 h with agitation. Digestion was then stopped by addition of HBB buffer (2% FBS and 0.2% BSA in HBSS) and filtered through a 40-μm filter into a 50-ml conical tube, transferred to a clean 15-ml conical tube and centrifuged at 350*g* for 5 min at 4 °C. Supernatant was then removed and pellet resuspended in 1 ml ACK lysing buffer (Gibco, A10492) and incubated at room temperature for 5 min followed by the addition of 9 ml DMEM. Suspension was then centrifuged under the above conditions, followed by removal of supernatant and resuspension in 5 ml FACS buffer (2% FBS and 2 mM EDTA in calcium/magnesium-free PBS). Centrifugation was repeated under the above conditions, the supernatant was removed and the pellet was resuspended in 300 μl cell resuspension buffer (0.04% BSA in 1× PBS) and 1 μl each of DRAQ5 (Thermo Fisher Scientific, 62251) and 4,6-diamidino-2-phenylindole (DAPI; BD Biosciences, 564907) and allowed to incubate for 5 min before sorting. DRAQ5^+^/DAPI^−^ cells were collected in cell resuspension buffer. Collected cells were then re-centrifuged according to the above parameters and resuspended in cell resuspension buffer to a target concentration of 1,000 cells μl^−1^. Cells were counted on a hemocytometer and the concentration was adjusted as necessary.

### Sample preparation for snRNA-seq.

Frozen cardiac tissues from LVAD cores or identical region from the apex of explanted donors were minced with a razor blade and transferred into a small (5 ml) Dounce homogenizer containing 1–2 ml of chilled lysis buffer (10 mM Tris-HCl, pH 7.4, 10 mM NaCl, 3 mM MgCl_2_ and 0.1% NP-40 in nuclease-free water). Samples were homogenized gently using five passes without rotation, then incubated on ice for 15 min. Lysate was then gently filtered through a 40-μm filter into 50-ml conical tube, followed by rinsing the filter once with 1 ml lysis buffer and transfer of lysate to a new 15-ml conical tube. Nuclei were then centrifuged at 500*g* for 5 min at 4 °C, followed by resuspension in 1 ml Nuclei Wash Buffer (2% BSA and 0.2 U μl^−1^ RNase inhibitor in 1× PBS) and filtered through a 20-μm pluristrainer into a fresh 15-ml conical tube. Centrifugation was repeated according to the above parameters. Supernatant was then removed and nuclei were resuspended in 300 μl Nuclei Wash Buffer and transferred to a 5-ml tube for flow sorting. Then, 1 μl DRAQ5 (5 mM solution; Thermo Fisher, cat. no. 62251) was added, mixed gently and allowed to incubate for 5 min before sorting. DRAQ5^+^ nuclei were sorted into Nuclei Wash Buffer on a BD FACS Melody (BD Biosciences) using a 100-μM nozzle. Recovered nuclei were centrifuged again under the above parameters and were gently resuspended in Nuclei Wash Buffer to a target concentration of 1,000 nuclei μl^−1^. Nuclei were counted on a hemocytometer and concentration was adjusted as necessary.

### sc/snRNA-seq analysis.

Cells and nuclei were processed using the Chromium Single Cell 5′ Reagent V1.1 kit from 10X Genomics. A total of 10,000 cells or nuclei per sample were loaded into a Chip G for GEM generation. Reverse transcription, barcoding, complementary DNA amplification and purification for library preparation were performed according to the Chromium 5′ V1.1 protocol. Sequencing was performed on a NovaSeq 6000 platform (Illumina) targeting 100,000 reads per cell or nucleus. Cells were aligned to the human GRCh38 transcriptome and nuclei were aligned to the whole genome pre-MRNA reference generated from the GRCh38 transcriptome using the CellRanger V3 software (10X Genomics) according to the 10X Genomics’ instructions. Filtering, unsupervised clustering, differential expression and additional analysis were completed using R and Python, including Seurat V3 and V4 and ClusterProfiler packages for R and the Palantir Python package.^24,68-70^

### QC, filtering and clustering.

For independent cell and nuclei analyses, individual sample matrices were imported into the Seurat v.3.2.3 R package and combined into a Seurat object. Cells were filtered for mitochondrial reads <10% and 2,000 < nCount_RNA < 10,000. Nuclei were filtered for mitochondrial reads <5% and 1,000 < nCount_RNA < 10,000. No filtering was applied based on nFeature_RNA. The objects were then saved for easy import after manual doublet removal. For each object, transformation and normalization was performed using SCTransform to fit a negative binomial distribution and regress out mitochondrial read percentage. Principle components (PCs) were then calculated (60 PCs for cells and 80 PCs for nuclei) and an elbow plot generated to select the cutoff for significant PCs to use for downstream analysis. UMAP dimensional reduction was then computed using the selected significant PCs (40 for cells and 80 for nuclei). Unsupervised clustering was then performed using the FindNeighbors and FindClusters function, again using the selected significant PC level as above, calculating clustering at a range of resolutions between 0.01–1. Differential gene expression was performed using the FindAllMarkers command using default parameters at high clustering resolution to aid in manual doublet discovery.

We utilized a supervised doublet removal method. Criteria to annotate cells as doublets included (1) high unique molecular identifier (UMI) counts and (2) gene expression signatures of two or more cell populations. Doublets often appear as clusters expressing markers of multiple cell populations within the dataset that overlapped with expression of nearby clusters^71-74^. Identification of doublet clusters was performed by generating *z* score expression profiles of each major cell population and plotting these signatures as well as UMI counts using UMAP/*t*-distributed stochastic neighbor embedding projections and heat maps. Cells annotated as doublets were removed and the list of remaining cells was saved. Raw objects from above were then loaded, subset to include cells that remained after doublet removal and clustering was repeated, starting with transformation and normalization. The supervised doublet removal process was repeated twice for the cell object and three times for the nuclei object until no doublet clusters were apparent.

To substantiate our supervised doublet removal method and compare our strategy to other doublet removal techniques, we ran Scrublet on our raw dataset. Using a Scrublet score of >0.2 (default setting) to identify doublets, we directly compared methodologies. We found a high concordance between cells annotated as doublets (86.7%) and cells retained in the final dataset (98.4%). Cell clusters identified as doublets using our supervised method corresponded to cells with a Scrublet score of >0.2. Furthermore, within the final integrated dataset analyzed in the manuscript, we did not identify any specific clusters that were composed of cells with high Scrublet scores (Supplementary Figs. 1 and 2).

Final resolutions used for analysis were selected following detection of DEGs at multiple resolutions and identifying the highest resolution at which significantly enriched genes were still present in each cluster (final resolution used was 0.6 for cell object and 0.5 for nuclei object). Metadata for condition, age, sex and cell type name were also added to the final objects.

### Integration of single-cell and single-nuclei datasets.

Integration of single-cell and single-nuclei datasets was performed using the R package, Harmony^75^. Filtered and SCTransformed objects from the single-cell and nucleus datasets were merged using the Seurat merge command and the RunHarmony command was then used to generate harmonized dimension reduction components using sequencing technology as the grouping variable. As recommended, we utilized 80 Harmony dimensions equal to the 80 PCA dimensions utilized in the single-nuclei dataset for performing re-clustering using the FindNeighbors and FindClusters Seurat commands at multiple resolutions between 0.1–1. No doublet exclusion or filtering was necessary as mapping and integration was performed on already filtered objects. The final resolution was selected to be 0.3 as this resolution captured the distinct cell types identified in the single-cell and nucleus datasets to be used for further analysis. Metadata for condition, age, sex and cell type name were also added to the final object.

Effectiveness of integration was evaluated by calculation of iLISI (integration local inverse Simpson’s index) scores using the R package, lisi^75,76^. The Harmony integration method was also compared to Seurat integration and reference mapping software. iLISI scores range from 1 (poor integration) to 2 (perfect integration). The iLISI scores for the three methods tested were, Harmony: 1.60, Seurat integration: 1.23 and Seurat reference mapping: 1.07, indicating high levels of integration using Harmony compared to other methods.

### Detection of differentially expressed genes.

Detection of DEGs between clusters was performed using the FindAllMarkers command, specifying return of only upregulated genes with a log_2_FC cutoff of 0.1. For downstream analysis, DEGs were further filtered by log_2_FC and *P* value as described for that analysis. For individual cell types, differential expression comparing only two groups by condition, sex or age was performed using the FindMarkers function specifying no minimum percentage of cells expressing an individual gene, return of both positively and negatively changed genes and no cutoffs for log_2_FC or *P* value to obtain even nonsignificant changes in expression for every gene present in the analysis. Filtering of this DEG table was performed by log_2_FC and *P* value for further analysis as described in the manuscript. For all DEG calculations the default ‘SCT’ assay and ‘data’ slot were used and performed using the default Wilcoxon rank-sum method. Results are presented for all major cell types observed (Supplementary Table 27).

### Calculation of population *z* scores.

*Z* score values were calculated using R v.3.6.2 and v.4.0.1. For each population where *z* scores were calculated, gene sets used were selected based on high enrichment in a population based on the DEG analysis described above. The expression matrix used to calculate *z* scores was extracted from a Seurat object using the GetAssayData function from the Seurat package from the default ‘SCT’ assay and ‘data’ slot. *Z* scores were then calculated for each gene set for each individual cell or nuclei in the dataset by scaling gene expression within the matrix, setting NA values introduced by conversion from a sparse matrix to 0 and using the following formula (no. of cells in dataset + sum of expression of genes in gene set) / no. of genes in gene set.

These calculated *z* scores were appended to a table to be saved as well as each *z* score added as metadata to the Seurat object for use in making feature plots.

### Pseudobulk RNA-seq.

Pseduobulk RNA-seq analysis was performed using the DESeq2 package for R. A gene expression matrix was extracted from the Seurat object using the GetAssayData Seurat function specifying the ‘RNA’ assay and ‘counts’ slot to extract raw sequencing counts for each gene and cell. Counts in this matrix were then summed per gene for each sample into a new matrix. The resulting matrix was normalized using DESeq2 by estimating size factors and performing normalization with the counts function, resulting in a new matrix with normalized counts for each gene and sample similar to the output of a traditional bulk sequencing experiment. The DESeq function was then utilized to calculate differential gene expression based on negative binomial distribution. Pairwise comparisons were completed by condition of interest (disease state, sex and age group) using the Wald test and an *α* value of 0.5 for independent filtering and adding log_2_FC using the lfcShrink function with ‘ashr’ adaptive shrinking. We specified no cutoffs for log_2_FC or *P* value to obtain even nonsignificant changes in expression. Filtering of this DEG table was performed by logFC and *P* value for further analysis as described in the manuscript.

### Analysis of associations with age.

Using cell type identities from the single-nuclei dataset, we aggregated counts and metadata to the sample level (split into donor and DCM separately) for each subject within each cell population and utilized DESeq2 to normalize the data using median of ratios to normalize counts and a regularized log transform of the normalized counts. We then used the normalized counts matrix to calculate Pearson correlation coefficients using the scipy stats function pearsonr to measure the linear relationship between each gene and age. Using genes with a Pearson correlation coefficient > ∣0.6∣ and *P* value <0.05, we constructed positive (Pearson coefficient >0.6) and negative (Pearson coefficient <−0.6) age-associated gene set *z* scores. We used the scipy stats linregress package in Python to perform linear regression analysis on the positive and negative aging signature as a function of age.

### Pathway analysis.

Pathway analysis was completed using the ClusterProfiler R package. A list of genes present in both the Seurat and Pseudobulk differential expression analyses by disease state with log_2_FC > 0.1 and adjusted *P* value <0.05 was utilized in the pathway analysis (Supplementary Fig. 3). Genes with negative and positive log_2_FC values were separated to identify enrichment in either the non-diseased or diseased condition, respectively. The enrichWP function was used to return a table with pathway enrichments from the WikiPathways database.

For comparison of enriched pathways between multiple populations/states, the compareCluster function was utilized on a matrix from the output Seurat differential expression analysis filtered for log_2_FC > 0.1 and adjusted *P* value <0.05 that contained the column specifying in which population/state the gene was upregulated. This analysis utilized the enrichPathway database from ClusterProfiler to return a table of enriched pathways in each population/state.

### Transcription factor analysis.

Transcription factor analysis was performed using the Enrichr web utility (https://maayanlab.cloud/Enrichr/enrich). Genes upregulated in a population/state based on Seurat differential expression analysis filtered for log_2_FC > 0.1 and adjusted *P* value <0.05 (Supplementary Fig. 3) were entered into the Enrichr and results from enrichment in the ChEA 2016 ChIP-seq database were downloaded and loaded as a matrix in R v.4.0.3 for the generation of dot plots.

### Trajectory analysis.

Trajectory analysis was performed using the Palantir package for Python. Using the normalized and scaled gene counts for the 3,000 highly variable genes, a matrix was exported as the input. Using the matrix, PCs were calculated and then diffusion maps were calculated as an estimate of the low dimensional phenotypic manifold of the data. Then, the actual Palantir was run by specifying a start cell state (the progenitor cell type from the dataset). Palantir then returned the terminal cell states, entropy values, pseudotime values and the probability of ending up in each of the terminal states for all cells.

### RNAScope in situ hybridization.

RNA was visualized using RNAScope Multiplex Fluorescent Reagent kit v2 Assay, RNAScope 2.5 HD Detection Reagent – RED and RNAScope 2.5 HD Duplex Assay kits (Advanced Cell Diagnostics, ACDBio) using probes designed by Advanced Cell Diagnostics for ANKRD1, MYH6, NPPA, NPPB, CD163, DCN, POSTN, PLA2G2A, CCL2, PCOLCE2, ELN and RGS5 (ref. ^77^). Samples were fixed for 24 h at 4 °C in 10% neutral buffered formalin. Samples were washed in 1× PBS, equilibrated in 30% sucrose, embedded in OCT medium (Sakura Finetek) and stored at −80 °C (fluorescence) or washed in 1× PBS, dehydrated in ethanol and embedded in paraffin (red and duplex). OCT-embedded sections were cut at 12 μm and paraffin-embedded sections were cut at 8 μm. Fluorescent images were collected using a Zeiss LSM 700 laser scanning confocal microscope. Chromogenic/brightfield images were acquired using a Zeiss Axioscan Z1 automated slide scanner. Image processing was performed using Zen Blue and Zen Black (Zeiss), FIJI/ImageJ^78,79^ and Photoshop (Adobe). The following RNAScope probes produced by ACDBio were utilized: ANKRD1 (524241), MYH6 (555381), NPPA (531281), NPPB (448511), CD163 (417061), DCN (589521), POSTN (409181), PLA2G2A (581101), CCL2 (423811), PCOLCE2 (566861), RGS5 (533421), ELN (408261), ACKR1 (525131), BTNL9 (430351) and CCL21 (474371).

## Extended Data

## Supplementary Material

supplement

Supplemental Table 20

Supplemental Table 21

Supplemental Table 22

Supplemental Table 23

Supplemental Table 24

Supplemental Table 25

Supplemental Table 26

Supplemental Table 27

## Data Availability

The processed single-cell objects, raw expression matrices and raw sequence files that support the findings of this study are available on the Gene Expression Omnibus (GSE183852). Alignment was performed to the publicly available transcriptome GRCh38-1.2.0.
