# Supplementary material for: Single-cell transcriptomics reveals cell-type-specific diversification in human heart failure
Source: Nat Cardiovasc Res. Author manuscript; Available in PMC 2022 Aug 10. (PMC9364913; doi:10.1038/s44161-022-00028-6)
Supplement: supplement [file NIHMS1824360-supplement-supplement.pdf]

nature portfolio

https://doi.org/10.1038/s44161-022-00028-6

Supplementary information

Single-cell transcriptomics reveals cell-type-specific diversification in human heart failure

In the format provided by the authors and unedited

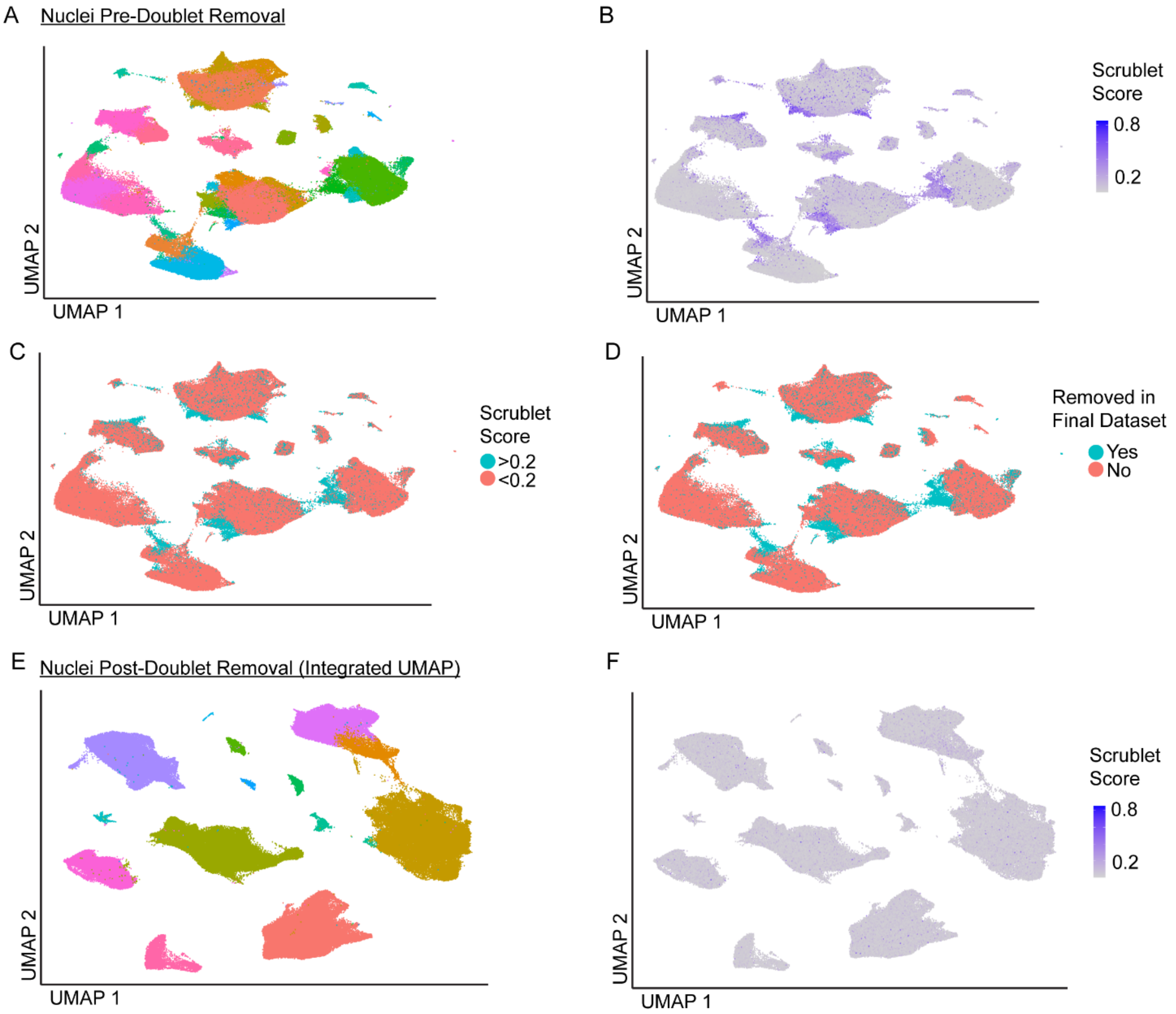

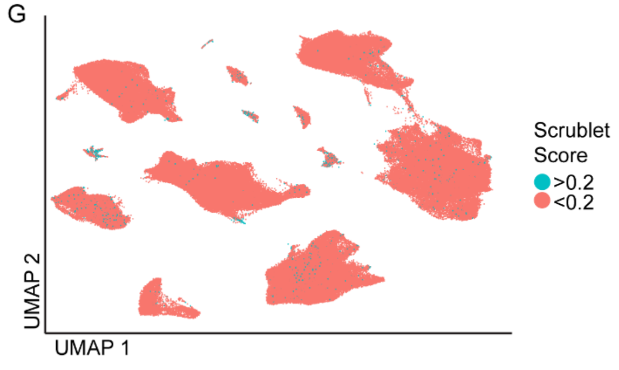

H

Doublet Removal Statistics	
Condition	# Nuclei
Pre-Doublet Removal	254590
Nuclei w/ scrublet score>0.2	25702
Nuclei removed	33838
Correlation between methods (removed)	22273/25702 (86.7%)
Retained after scrublet score	228888
Retained in final dataset	220752
Correlation between methods (retained)	217322/220752 (98.4%)

Supplementary Figure 1. Comparison of doublet removal strategies.

A, UMAP projection of single nucleus RNA sequencing data pre-doublet removal. **B**, Scrublet scores projected on the pre-doublet removal data. **C-D**, UMAP projection of single nucleus RNA sequencing data pre-doublet removal demonstrating correspondence of cells with a Scrublet score >0.2 (C) and cells removed using our supervised doublet protocol (D). **E**, UMAP projection of the final integrated single nucleus/cell RNA sequencing dataset following supervised doublet removal. **F,** Scrublet scores projected on the final integrated single nucleus/cell RNA sequencing dataset. **G**, UMAP projection of the final integrated single nucleus/cell RNA sequencing dataset following supervised doublet removal indicating cells with a Scrublet score >0.2. **H**, Data table comparing Scrublet and supervised double removal approaches.

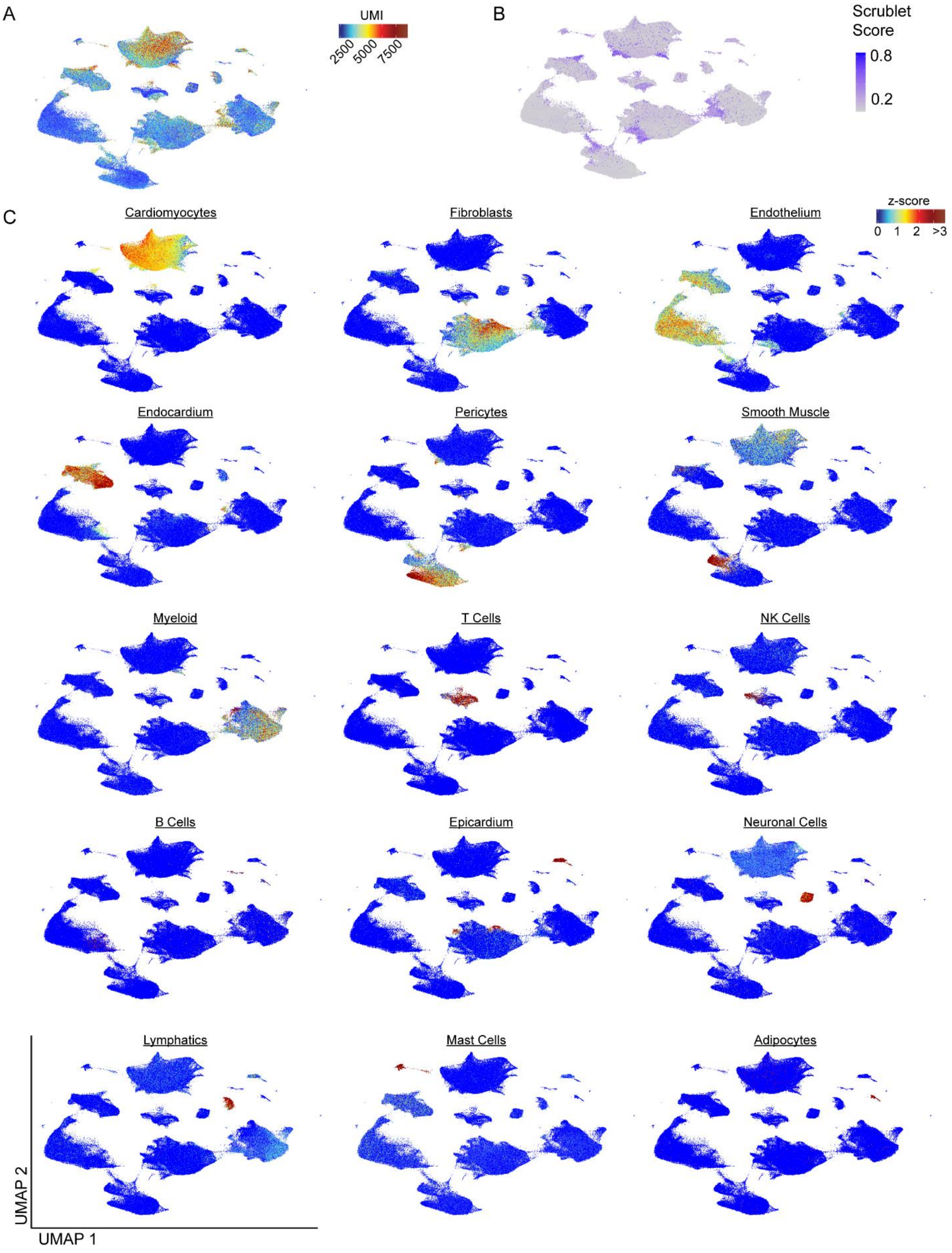
A
UMI
2500
5000
7500
B
Scrublet Score
0.8
0.2
C
z-score
0
1
2
>3
Cardiomyocytes
Fibroblasts
Endothelium
Endocardium
Pericytes
Smooth Muscle
Myeloid
T Cells
NK Cells
B Cells
Epicardium
Neuronal Cells
Lymphatics
Mast Cells
Adipocytes
UMAP 2
UMAP 1

Supplementary Figure 2. Supervised doublet removal protocol.

A, UMI counts projected on single nucleus RNA sequencing data pre-doublet removal. **B**, Scrublet scores projected on single nucleus RNA sequencing data pre-doublet removal. **C**, Gene expression signatures for major cell populations projected on the single nucleus RNA sequencing data pre-doublet removal. Criteria for doublets included increased UMI count and the presence of 2 or more overlapping major cell population gene expression signatures.

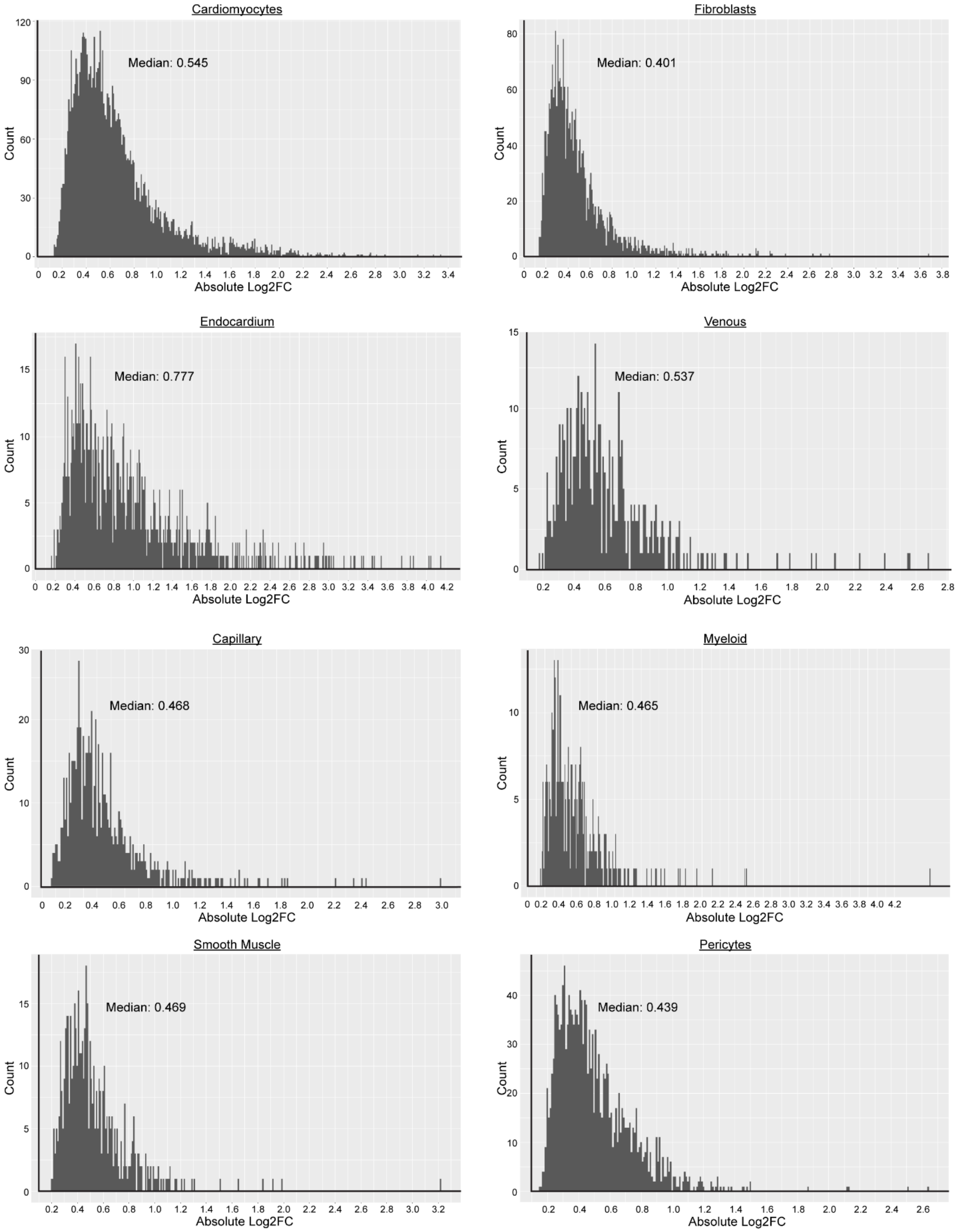

Cardiomyocytes
Median: 0.545
Count
Absolute Log2FC
Fibroblasts
Median: 0.401
Count
Absolute Log2FC
Endocardium
Median: 0.777
Count
Absolute Log2FC
Venous
Median: 0.537
Count
Absolute Log2FC
Capillary
Median: 0.468
Count
Absolute Log2FC
Myeloid
Median: 0.465
Count
Absolute Log2FC
Smooth Muscle
Median: 0.469
Count
Absolute Log2FC
Pericytes
Median: 0.439
Count
Absolute Log2FC

Supplementary Figure 3. Distribution of genes differentially expressed in donor versus DCM samples.

Frequency histograms of genes used in pathway and transcription factor enrichment analyses. Genes derived from intersect of pseudobulk and Seurat differential expression analysis on nuclei with adjusted p-value <0.05 and Log2 fold change (FC) >0.1 in both datasets. The distribution (absolute Log2 FC) of genes differentially expressed between donor and DCM samples within each major cardiac cell population is displayed. Median Log2 FC values are annotated.

Supplementary Table 1. Demographic and Baseline Clinical Data Table for Combined Single-Nuclei and Single-Cell RNA Sequencing Analysis

	HEALTHY (N=27)	DCM (N=18)
**Demographics**		
Mean age (years)	50.2 ± 18.2	45.3 ± 19.5
Median age (years)	53	
Age range (years)	11 – 75	8 – 74
Sex – n. (%)		
Male	13 (46.4)	12 (66.7)
Female	15 (53.6)	6 (21.4)
Race – n. (%)		
White	20 (71.4)	12 (66.7)
Black or African American	8 (28.6)	6 (21.4)
Ethnicity – n. (%)		
Non-Hispanic	28 (100)	17 (94.4)
Hispanic	0 (0)	1 (5.6)
**Baseline Clinical Medical History**		
BMI (kg/m^2) – mean ± SD	28.8 ± 7.4	26.8 ± 7.2
Hypertension – n. (%)	16 (57.1)	8 (44.4)
Diabetes – no. (%)	9 (32.1)	6 (33.3)
Chronic Kidney Disease – n. (%)	2 (7.1)	7 (38.9)
Smoking – n. (%)	17 (60.7)	9 (50.0)
**Cardiac Clinical Data**		
Cardiac Ejection Fraction – mean ± SD	58.7 ± 11.1	19.8 ± 6.9
Cardiac Output (L/min) – mean ± SD	7.7 ± 2.4	3.2 ± 1.3
Arrhythmias – n. (%)	4 (14.3)	13 (72.2)
Valve Disease (≥mild) – n. (%)	8 (28.6)	14 (77.8)
Mitral valve	6 (21.4)	13 (72.2)
Aortic valve	3 (10.7)	2 (11.1)
Pulmonary valve	1 (3.6)	3 (16.7)
Tricuspid valve	5 (17.9)	8 (44.4)

Patient level metadata is available in Data Table D1

Supplementary Table 2. Post-QC metrics of single nucleus RNA sequencing data

Nuclei	nCount		nFeature		Mitochondria (%)	
	average	std. dev.	average	std. dev.	average	std. dev.
Fibroblasts	2521.2	1269.4	1485.3	545.8	0.5	0.5
Cardiomyocytes	5093.0	2280.7	2030.2	696.3	0.3	0.4
Lymphatics	2608.1	1458.8	1557.2	626.7	0.7	0.7
Neural	1754.4	946.1	1096.2	395.8	0.7	0.5
Endothelium	1833.2	775.1	1183.5	364.5	0.7	0.5
Epicardium	4163.3	1927.0	1900.3	655.9	0.4	0.6
Mast cells	2256.1	1394.0	1370.4	624.1	0.4	0.3
Adipocytes	5349.9	2312.3	2439.8	856.6	0.2	0.3
Macrophages	2358.2	1158.3	1441.2	512.0	0.5	0.5
Monocytes	1781.8	690.5	1187.2	337.9	1.3	1.0
Pericytes	1790.6	659.0	1098.5	315.3	0.7	0.6
B-cells	1976.6	974.0	1240.3	469.6	0.6	0.5
Endocardium	2743.1	1266.6	1577.2	517.7	0.6	0.6
Smooth muscle	1972.7	872.5	1147.3	372.0	0.4	0.4
T/NK-cells	1452.6	426.2	1024.2	233.6	0.9	0.7

std. dev.: standard deviation

Supplementary Table 3. Post-QC metrics of single cell RNA sequencing data

Cells	nCount		nFeature		Mitochondria (%)	
	average	std. dev.	average	std. dev.	average	std. dev.
Fibroblasts	5865.1	2248.4	2267.0	606.6	4.4	1.9
Endothelium	3563.3	1471.1	1666.1	441.9	3.8	2.0
Pericytes	3782.2	1297.3	1788.9	411.8	6.6	1.9
Macrophages	5191.8	2192.4	1688.1	488.9	5.7	1.9
T-cells	4041.5	1389.2	1509.2	402.6	5.0	1.7
Monocytes	5482.0	2198.2	1686.1	503.2	5.0	1.6
Smooth muscle	5403.2	1981.5	2129.7	524.7	7.7	1.7
NK-cells	3971.2	1530.6	1675.1	492.8	5.5	1.8
Neural	3508.5	1438.5	1820.7	505.0	4.9	1.8
B-cells	5000.6	2097.1	1481.1	565.5	5.2	2.3

std. dev.: standard deviation

Supplementary Table 4. Comparison of differentially expressed genes in the single nucleus RNA sequencing dataset identified by Seurat vs. pseudobulk analysis.

	Seurat	Pseudobulk	Overlap
Cardiomyocytes	6457	6280	4418
Smooth muscle	613	809	443
Fibroblasts	7793	6342	4666
Myeloid	7359	3998	2125
Pericytes	2851	3090	1751
Endothelium	4424	4106	2566
Endocardium	4514	3753	3046
T/NK-cells	533	109	99

Only major cell populations are shown.

Supplementary Table 5. Comparison of differentially expressed genes in the single cell RNA sequencing dataset identified by Seurat vs. pseudobulk analysis.

	Seurat	Pseudobulk	Overlap
Fibroblasts	5298	1758	1720
Myeloid	1531	244	198
Pericytes	876	54	49
Endothelium	2230	281	279
T/NK-cells	652	167	100
Smooth muscle	157	1	1

Only major cell populations are shown.

Supplementary Table 6. Cell composition as a function of disease.

	Donor	DCM	p-value
Cardiomyocytes	25.7 (14)	10.0 (3.4)	1.25E-05
Smooth muscle	2.1 (0.7)	2.5 (0.8)	2.44E-01
Fibroblasts	18.3 (4.5)	29.1 (5.1)	3.92E-06
Endothelium	16.6 (5.7)	17.9 (5.3)	5.04E-01
Lymphatic	0.8 (0.7)	1.7 (1.1)	2.80E-02
Neural	0.7 (0.4)	0.8 (0.4)	6.48E-01
Epicardium	0.7 (1.1)	1.1 (2.8)	6.64E-01
Mast	0.7 (0.6)	0.3 (0.3)	3.31E-03
B-Cells	0.1 (0.1)	0.1 (0.2)	2.42E-01
Adipocytes	0.5 (0.9)	0.4 (0.4)	6.79E-01
Myeloid	12.8 (4.4)	18.8 (6.7)	1.20E-02
Pericytes	13.1 (5.6)	6.1 (1.5)	2.66E-06
Endocardium	6.2 (4.8)	7.8 (9.6)	5.77E-01
T/NK-cells	1.6 (1.2)	3.4 (2.4)	2.43E-02

Data shown as a percentage of total cell types. Standard deviations are provided in parentheses. $p<0.05$ highlighted in red. p-values calculated by Welch's two-tailed T-Test.

Supplementary Table 7. Cell composition in female hearts as a function of disease.

Female			
	Donor	DCM	p-value
Cardiomyocytes	25.7 (15)	9.6 (2.4)	2.52E-03
Smooth muscle	2.2 (0.7)	2.4 (0.8)	5.55E-01
Fibroblasts	16.8 (5.1)	28.1 (4.9)	1.68E-03
Endothelium	18.3 (5.5)	20.8 (5.9)	4.39E-01
Lymphatic	1.0 (0.9)	1.7 (0.9)	1.57E-01
Neural	0.8 (0.4)	0.6 (0.3)	3.04E-01
Epicardium	0.7 (1.2)	0.5 (0.7)	6.74E-01
Mast	0.7 (0.6)	0.2 (0.1)	2.20E-02
B-Cells	0.1 (0.1)	0.1 (0.1)	5.45E-01
Adipocytes	0.5 (1.1)	0.4 (0.2)	6.19E-01
Myeloid	12.2 (5.1)	21.5 (6.6)	2.22E-02
Pericytes	12.6 (4.6)	6.0 (1.7)	4.57E-04
Endocardium	6.5 (4.6)	5.5 (4.2)	6.69E-01
T/NK-cells	1.8 (1.2)	2.5 (1.5)	3.72E-01

Data shown as a percentage of total cell types. Standard deviations are provided in parentheses. p<0.05 highlighted in red. p-values calculated by Welch's two-tailed T-Test.

Supplementary Table 8. Cell composition in male hearts as a function of disease.

Male			
	Donor	DCM	p-value
Cardiomyocytes	25.6 (13)	10.5 (4)	2.88E-03
Smooth muscle	2.1 (0.7)	2.5 (0.7)	3.22E-01
Fibroblasts	19.8 (3.0)	29.9 (5.2)	2.41E-03
Endothelium	14.6 (5.3)	15.3 (2.9)	7.36E-01
Lymphatic	0.7 (0.5)	1.6 (1.2)	1.16E-01
Neural	0.7 (0.4)	1.0 (0.4)	1.67E-01
Epicardium	0.8 (1.0)	1.6 (3.7)	5.96E-01
Mast	0.8 (0.5)	0.4 (0.4)	6.74E-02
B-Cells	0.1 (0.1)	0.2 (0.3)	3.26E-01
Adipocytes	0.5 (0.5)	0.5 (0.6)	9.42E-01
Myeloid	13.4 (3.4)	16.5 (5.9)	2.79E-01
Pericytes	13.7 (6.5)	6.2 (1.3)	2.49E-03
Endocardium	5.8 (4.9)	9.9 (12)	4.58E-01
T/NK-cells	1.4 (1.1)	4.2 (2.7)	4.42E-02

Data shown as a percentage of total cell types. Standard deviations are provided in parentheses. p<0.05 highlighted in red. p-values calculated by Welch's two-tailed T-Test.

Supplementary Table 9. Cell composition in DCM hearts as a function of INTERMACS score.

DCM			
	INTERMACS 1-2	INTERMACS 3-4	p-value
Cardiomyocytes	12.2 (3.1)	8.7 (2.8)	0.09
Smooth muscle	2.0 (0.5)	2.7 (0.8)	0.10
Fibroblasts	28.7 (6.6)	29.3 (4.0)	0.88
Endothelium	16.8 (3.2)	18.5 (6.2)	0.55
Lymphatic	1.5 (0.7)	1.8 (1.2)	0.59
Neural	0.8 (0.3)	0.8 (0.4)	1.00
Epicardium	0.1 (0.1)	1.8 (3.4)	0.24
Mast	0.2 (0.2)	0.3 (0.3)	0.50
B-Cells	0.0 (0.1)	0.2 (0.3)	0.13
Adipocytes	0.6 (0.6)	0.3 (0.3)	0.51
Myeloid	19.1 (7.1)	18.6 (6.4)	0.91
Pericytes	5.2 (0.3)	6.6 (1.7)	0.06
Endocardium	9.7 (9.7)	6.7 (9.3)	0.63
T/NK-cells	3.0 (1.4)	3.7 (2.8)	0.61

Data shown as a percentage of total cell types. Standard deviations are provided in parentheses. p-values calculated by Welch's two-tailed T-Test.

Supplementary Table 10. Cell composition in donor hearts as a function of sex.

Donor			
	Male	Female	p-value
Cardiomyocytes	25.6 (13)	25.7 (14)	0.99
Smooth muscle	2.1 (0.7)	2.2 (0.7)	0.77
Fibroblasts	19.8 (3.0)	16.8 (5.1)	0.10
Endothelium	14.6 (5.3)	18.3 (5.5)	0.11
Lymphatic	0.7 (0.5)	1.0 (0.9)	0.48
Neural	0.7 (0.4)	0.8 (0.4)	0.42
Epicardium	0.8 (1.0)	0.7 (1.2)	0.98
Mast	0.8 (0.5)	0.7 (0.6)	0.92
B-Cells	0.1 (0.1)	0.1 (0.1)	0.92
Adipocytes	0.5 (0.5)	0.5 (1.1)	0.89
Myeloid	13.4 (3.4)	12.2 (5.1)	0.49
Pericytes	13.7 (6.5)	12.6 (4.6)	0.65
Endocardium	5.8 (4.9)	6.5 (4.6)	0.74
T/NK-cells	1.4 (1.1)	1.8 (1.2)	0.38

Data shown as a percentage of total cell types. Standard deviations are provided in parentheses. p-values calculated by Welch's two-tailed T-Test.

Supplementary Table 11. Cell composition in DCM hearts as a function of sex.

DCM			
	Male	Female	p-value
Cardiomyocytes	10.5 (4.0)	9.6 (9.6)	0.66
Smooth muscle	2.5 (0.7)	2.4 (2.4)	0.95
Fibroblasts	29.9 (5.2)	28.1 (4.9)	0.58
Endothelium	15.3 (2.9)	20.8 (5.9)	0.10
Lymphatic	1.6 (1.2)	1.7 (0.9)	0.94
Neural	1.0 (0.4)	0.6 (0.3)	0.13
Epicardium	1.6 (3.7)	0.5 (0.7)	0.52
Mast	0.4 (0.4)	0.2 (0.1)	0.50
B-Cells	0.2 (0.3)	0.1 (0.1)	0.48
Adipocytes	0.5 (0.6)	0.4 (0.2)	0.70
Myeloid	16.5 (5.9)	21.5 (6.6)	0.22
Pericytes	6.2 (1.3)	6.0 (1.7)	0.85
Endocardium	9.9 (12.1)	5.5 (4.2)	0.43
T/NK-cells	4.2 (2.7)	2.5 (1.5)	0.24

Data shown as a percentage of total cell types. Standard deviations are provided in parentheses. p-values calculated by Welch's two-tailed T-Test.

Supplementary Table 12. Regression of cell composition in donor hearts as a function of age.

Donor	All		Male		Female	
	R	p-value	R	p-value	R	p-value
Cardiomyocyte	0.19	5.32E-01	0.03	9.40E-01	0.50	3.16E-01
Fibroblast	0.15	6.22E-01	0.70	8.11E-02	-0.38	4.54E-01
Endothelium	-0.24	4.31E-01	-0.56	1.90E-01	0.50	3.10E-01
Endocardium	-0.51	7.55E-02	-0.34	4.53E-01	-0.77	7.35E-02
Smooth muscle	0.16	6.04E-01	0.50	2.48E-01	-0.13	8.12E-01
Perciyte	-0.29	3.37E-01	0.22	6.41E-01	-0.67	1.44E-01
Myeloid	0.68	1.11E-02	0.51	2.40E-01	0.89	1.63E-02
Lymphatic	0.08	7.98E-01	-0.02	9.69E-01	0.21	6.96E-01
Neural	-0.20	5.22E-01	0.31	5.04E-01	-0.86	2.98E-02
Epicardium	0.07	8.11E-01	0.27	5.55E-01	-0.78	6.72E-02
Mast	0.09	7.67E-01	0.08	8.64E-01	0.20	7.03E-01
Adipocyte	0.23	4.54E-01	0.55	2.03E-01	-0.45	3.74E-01
T/NK-cells	-0.42	1.57E-01	-0.42	3.46E-01	-0.52	2.88E-01

R: regression coefficient. Positive value indicates a correlation with increasing age. Negative value indicates a correlation with decreasing age. $p<0.05$ highlighted in red. R and p-values calculated by two-tailed linear regression Wald test with t-distribution.

Supplementary Table 13. Regression of cell composition in DCM hearts as a function of age.

DCM	All		Male		Female	
	R	p-value	R	p-value	R	p-value
Cardiomyocyte	-0.20	3.41E-01	-0.24	4.50E-01	-0.17	5.77E-01
Fibroblast	-0.08	7.16E-01	-0.04	9.08E-01	-0.02	9.57E-01
Endothelium	0.11	6.07E-01	0.25	4.30E-01	-0.05	8.70E-01
Endocardium	0.03	8.69E-01	-0.15	6.46E-01	0.09	7.66E-01
Smooth muscle	0.27	1.88E-01	0.30	3.42E-01	0.23	4.45E-01
Perciyte	0.10	6.27E-01	0.27	3.98E-01	-0.06	8.42E-01
Myeloid	0.21	3.11E-01	0.32	3.18E-01	0.20	5.11E-01
Lymphatic	0.17	4.09E-01	-0.63	2.91E-02	0.56	4.47E-02
Neural	-0.22	2.88E-01	-0.36	2.51E-01	-0.16	6.06E-01
Epicardium	0.11	6.00E-01	0.22	5.01E-01	0.03	9.26E-01
Mast	0.07	7.36E-01	-0.15	6.49E-01	0.23	4.47E-01
Adipocyte	0.22	2.80E-01	0.09	7.89E-01	0.30	3.20E-01
T/NK-cells	0.22	2.88E-01	-0.01	9.63E-01	0.37	2.19E-01

R: regression coefficient. Positive value indicates a correlation with increasing age. Negative value indicates a correlation with decreasing age. $p<0.05$ highlighted in red. R and p-values calculated by two-tailed linear regression Wald test with t-distribution.

Supplementary Table 14. Regression of gene expression in donor hearts as a function of age (transcripts enriched in young patients).

Donor	All		Male		Female	
	R	p-value	R	p-value	R	p-value
Cardiomyocyte	-0.91	2.46E-10	-0.92	2.40E-05	-0.92	8.73E-06
Fibroblast	-0.92	5.47E-11	-0.93	9.84E-06	-0.91	1.27E-05
Endocardium	-0.92	4.44E-11	-0.92	2.46E-05	-0.93	2.85E-06
Endothelium	-0.93	2.29E-11	-0.92	1.87E-05	-0.93	3.11E-06
Smooth muscle	-0.90	1.14E-09	-0.89	1.29E-04	-0.92	6.76E-06
Pericyte	-0.93	1.09E-11	-0.91	3.53E-05	-0.96	2.25E-07
Myeloid	-0.93	1.38E-11	-0.97	3.82E-07	-0.90	2.63E-05
Adipocytes	-0.88	4.58E-09	-0.86	3.13E-04	-0.90	2.71E-05
Lymphatic	-0.85	9.20E-08	-0.85	4.57E-04	-0.86	1.87E-04
Neural	-0.92	7.34E-11	-0.93	1.31E-05	-0.92	1.13E-05
Epicardium	-0.77	1.03E-05	-0.82	1.79E-03	-0.71	6.63E-03
Mast	-0.92	6.97E-11	-0.93	1.22E-05	-0.92	8.93E-06
T/NK-Cells	-0.78	4.78E-06	-0.78	3.02E-03	-0.78	1.48E-03

R: regression coefficient. Positive value indicates a correlation with increasing age. Negative value indicates a correlation with decreasing age. Pearson correlations were calculated for the full dataset and then individually tested in male and female subjects. R and p-values calculated by two-tailed linear regression Wald test with t-distribution.

Supplementary Table 15. Regression of gene expression in donor hearts as a function of age (transcripts enriched in older patients).

Donor	All		Male		Female	
	R	p-value	R	p-value	R	p-value
Cardiomyocyte	0.87	1.65E-08	0.85	4.74E-04	0.88	6.99E-05
Fibroblast	0.96	1.54E-14	0.97	1.08E-07	0.95	5.41E-07
Endocardium	0.97	5.34E-15	0.98	1.73E-08	0.96	1.11E-07
Endothelium	0.92	1.50E-10	0.93	1.46E-05	0.90	2.62E-05
Smooth muscle	0.88	5.10E-09	0.87	2.49E-04	0.89	4.12E-05
Pericyte	0.94	1.62E-12	0.96	3.96E-07	0.93	5.98E-06
Myeloid	0.93	1.71E-11	0.94	3.64E-06	0.92	7.29E-06
Adipocytes	0.74	2.53E-05	0.76	3.94E-03	0.73	4.67E-03
Lymphatic	0.81	1.14E-06	0.74	5.69E-03	0.89	5.16E-05
Neural	0.86	3.96E-08	0.86	3.11E-04	0.86	1.60E-04
Epicardium	0.89	5.27E-09	0.92	5.28E-05	0.86	1.69E-04
Mast	0.92	8.63E-11	0.93	1.35E-05	0.91	1.52E-05
T/NK-Cells	0.92	1.30E-10	0.96	9.33E-07	0.89	4.47E-05

R: regression coefficient. Positive value indicates a correlation with increasing age. Negative value indicates a correlation with decreasing age. Pearson correlations were calculated for the full dataset and then individually tested in male and female subjects. R and p-values calculated by two-tailed linear regression Wald test with t-distribution.

Supplementary Table 16. Regression of gene expression in DCM hearts as a function of age (transcripts enriched in young patients).

DCM	All		Male		Female	
	R	p-value	R	p-value	R	p-value
Cardiomyocyte	-0.97	3.83E-08	-0.96	4.44E-04	-0.98	8.10E-04
Fibroblast	-0.95	4.26E-07	-0.95	9.73E-04	-0.97	1.34E-03
Endocardium	-0.95	5.55E-07	-0.90	6.37E-03	-1.00	3.68E-05
Endothelium	-0.96	1.09E-07	-0.97	3.27E-04	-0.96	2.08E-03
Smooth muscle	-0.98	1.12E-09	-0.99	1.88E-05	-0.98	3.82E-04
Pericyte	-0.97	2.22E-08	-0.98	7.09E-05	-0.97	9.90E-04
Myeloid	-0.96	1.19E-07	-0.98	1.40E-04	-0.96	2.95E-03
Adipocytes	-0.96	9.01E-06	-0.99	9.50E-03	-0.97	1.75E-03
Lymphatic	-0.98	1.02E-08	-0.98	6.05E-05	-0.98	7.59E-04
Neural	-0.96	2.98E-07	-0.95	1.07E-03	-0.98	5.74E-04
Epicardium	-0.94	4.36E-06	-0.89	1.71E-02	-0.95	3.22E-03
Mast	-0.89	4.86E-05	-0.97	2.78E-04	-0.95	3.96E-03
T/NK-Cells	-0.97	2.65E-08	-0.96	5.87E-04	-1.00	5.74E-06

R: regression coefficient. Positive value indicates a correlation with increasing age. Negative value indicates a correlation with decreasing age. Pearson correlations were calculated for the full dataset and then individually tested in male and female subjects. R and p-values calculated by two-tailed linear regression Wald test with t-distribution.

Supplementary Table 17. Regression of gene expression in DCM hearts as a function of age (transcripts enriched in older patients).

DCM	All		Male		Female	
	R	p-value	R	p-value	R	p-value
Cardiomyocyte	0.97	6.29E-08	0.97	3.12E-04	0.97	1.37E-03
Fibroblast	0.96	2.81E-07	0.93	2.74E-03	0.99	1.98E-04
Endocardium	0.95	4.91E-07	0.92	3.83E-03	0.98	3.37E-04
Endothelium	0.98	1.92E-09	0.97	3.63E-04	1.00	1.30E-05
Smooth muscle	0.99	4.87E-10	0.99	4.45E-05	0.99	2.28E-04
Pericyte	0.97	3.02E-08	0.95	9.18E-04	0.99	5.22E-05
Myeloid	0.97	3.22E-08	0.97	2.97E-04	0.99	4.41E-05
Adipocytes	0.95	2.09E-05	0.87	1.28E-01	0.97	1.36E-03
Lymphatic	0.99	2.01E-11	1.00	1.41E-06	0.99	6.69E-05
Neural	0.94	1.25E-06	0.92	3.31E-03	0.97	1.09E-03
Epicardium	0.94	5.62E-06	0.89	1.65E-02	0.97	1.17E-03
Mast	0.95	1.07E-06	0.97	3.24E-04	0.93	6.49E-03
T/NK-Cells	0.79	1.37E-03	0.83	2.21E-02	0.83	4.10E-02

R: regression coefficient. Positive value indicates a correlation with increasing age. Negative value indicates a correlation with decreasing age. Pearson correlations were calculated for the full dataset and then individually tested in male and female subjects. R and p-values calculated by two-tailed linear regression Wald test with t-distribution.

Supplementary Table 18. Pathway analysis of genes associated with age in donor controls

Cell type	Direction	Pathway	Adj. p-value
Cardiomyocytes	younger	FOXA2 pathway	2.67E-02
Cardiomyocytes	older	NO/cGMP/PKG mediated neuroprotection	3.05E-02
Cardiomyocytes	younger	7q11.23 copy number variation syndrome	4.03E-02
Cardiomyocytes	younger	Angiopoietin-like protein 8 regulatory pathway	4.03E-02
Cardiomyocytes	younger	Nonalcoholic fatty liver disease	4.03E-02
Endocardium	older	MFAP5-mediated ovarian cancer cell motility and invasiveness	3.47E-02
Endocardium	older	Cell-type dependent selectivity of CCK2R signaling	3.47E-02
Endocardium	older	PKC-gamma calcium signaling pathway in ataxia	3.91E-02
Fibroblasts	older	OSX and miRNAs in tooth development	3.94E-02
Fibroblasts	younger	EDA signaling in hair follicle development	5.35E-03
Fibroblasts	younger	Spinal cord injury	2.29E-02
Fibroblasts	younger	IL-18 signaling pathway	3.55E-02
Myeloid	older	Cell-type dependent selectivity of CCK2R signaling	1.21E-02
Myeloid	older	SREBF and miR33 in cholesterol and lipid homeostasis	1.21E-02
older	older	Cannabinoid receptor signaling	1.23E-02
Myeloid	older	Sterol regulatory element-binding proteins (SREBP) signaling	2.31E-02
Myeloid	older	22q11.2 copy number variation syndrome	3.33E-02
Pericytes	older	Overview of leukocyte-intrinsic Hippo pathway functions	1.82E-02
Pericytes	older	Nucleotide-binding oligomerization domain (NOD) pathway	1.82E-02
Pericytes	older	IL-6 signaling pathway	1.82E-02
Pericytes	older	mRNA processing	4.20E-02
Pericytes	older	Vitamin D receptor pathway	4.70E-02
Pericytes	younger	7q11.23 copy number variation syndrome	1.35E-02
Smooth Muscle	older	Urea cycle and associated pathways	4.44E-02
Smooth Muscle	older	Renin-angiotensin-aldosterone system (RAAS)	4.44E-02
Smooth Muscle	younger	Fragile X syndrome	1.57E-02

p-value calculated using hypergeometric distribution and corrected for multiple comparisons.

Supplementary Table 19. Pathway analysis of genes associated with age in DCM

Cell type	Direction	Pathway	Adj. p-value
Cardiomyocytes	younger	Homologous recombination	2.40E-02
Cardiomyocytes	younger	DNA repair pathways, full network	2.40E-02
Endothelium	older	NOTCH1 regulation of endothelial cell calcification	7.27E-03
Endothelium	older	Embryonic stem cell pluripotency pathways	7.27E-03
Endocardium	younger	Cytoplasmic ribosomal proteins	4.49E-04
Fibroblasts	younger	Cytoplasmic ribosomal proteins	6.08E-07
Myeloid	older	Primary focal segmental glomerulosclerosis (FSGS)	1.27E-02
Myeloid	younger	Cytoplasmic ribosomal proteins	9.21E-04
Myeloid	younger	Genes associated with the development of rheumatoid arthritis	3.37E-02
T Cells	younger	Cytoplasmic ribosomal proteins	1.23E-04

p-value calculated using hypergeometric distribution and corrected for multiple comparisons.
